# Design and performance evaluation of a solar powered remote controlled robot for precision seed planting

**DOI:** 10.1038/s41598-026-68946-0

**Published:** 2026-09-15

**Authors:** Shimaa Aboharg, El-Keway Abdelfattah, Ismail Abdelmotaleb, Ayman Eldesoukey, Z. M. Omara, Noureldin Sharaby

**Affiliations:** 1 Agricultural Engineering Department, Faculty of Agriculture, Kafrelsheikh University, Kafrelsheikh, Egypt; 2 Agricultural Engineering Research Institute, Dokki, Giza, Egypt; 3 Mechanical Engineering Department, Faculty of Engineering, Kafrelsheikh University, Kafrelsheikh, Egypt

**Keywords:** Sustainability, Solar-powered robot, Precision planting, Sustainable agriculture, Electric seed metering, Remote-controlled agricultural robotics, Energy science and technology, Engineering

## Abstract

The increasing demand for sustainable agricultural mechanization has accelerated the development of precision and energy-efficient planting systems. This study presents the design, optimization, and performance evaluation of a solar-powered remotely controlled robot for precision seed planting. The robotic platform integrates a photovoltaic energy system, an electric seed-metering mechanism, and an electronically controlled seed-depth adjustment unit to achieve accurate seed placement while minimizing energy consumption. The system was experimentally evaluated at four robot forward speeds (0.42–1.60 km h^−1^) and four target seed spacings (10–25 cm). Results showed high seed placement accuracy ranging from 98.08% to 98.80%, while the miss and multiple indices remained below 2% and 2.5%, respectively. Optimal metering performance was obtained at robot speeds of 0.82–1.20 km h^−1^. The seed-depth adjustment mechanism exhibited a strong linear relationship between motor rotations and penetration depth (R^2^ = 0.9996), ensuring precise depth control. Energy analysis indicated low power consumption (0.048–0.084 kWh) and a solar power supply ratio (95–167%). These findings demonstrate that integrating solar energy with wirelessly controlled agricultural robotics can improve planting precision while enhancing energy efficiency and environmental sustainability in precision farming systems.

## Introduction

The global agri‑food system faces unprecedented challenges to boost productivity to meet the demands of a growing population, reduce environmental impacts, and address shrinking rural labor availability in many agricultural regions^1,2^. Significant increases in productivity and input-use efficiency are needed to feed a population that is expected to reach around 10 billion people by 2050, especially in smallholder and labor-constrained production systems^3^. Although traditional mechanization has increased agricultural productivity, it still mostly depends on fossil fuels, large equipment, and a lot of manual labor, which can worsen soil compaction, environmental degradation, greenhouse gas emissions, and economic barriers for small and medium-sized farms^4^. These challenges motivate a transition to automated, energy-efficient, and sustainable field operations.

Precision agriculture has emerged as a key technological approach for improving crop productivity, resource efficiency, and environmental sustainability in modern farming systems. By integrating sensing technologies, automation, and data-driven decision systems, precision agriculture enables optimized field operations and improved crop management practices^5–7^.

Among agricultural operations, precision seeding is one of the most critical stages influencing crop establishment, plant population uniformity, and final yield. Accurate seed placement in terms of spacing and depth ensures uniform plant growth and reduces competition for water, nutrients, and sunlight^8^. Conventional mechanical seed metering systems often depend on ground-driven mechanisms, which can cause slippage, vibration, and uneven seed distribution in shifting field conditions^9^. Under variable field conditions, these systems may experience synchronization errors between machine forward speed and seed dispensing frequency, resulting in irregular seed spacing, missed seeds, or multiple seed drops^10^. Precision planting applications can achieve more precise seed placement due to electrically driven seed metering systems, which enhance controllability and synchronization of forward speed and seed release time^11,12^.

To address these limitations, researchers have increasingly focused on the development of electrically driven seed-metering mechanisms. Electric drive systems allow independent control of the metering device through electronic controllers and sensors, enabling precise synchronization between forward motion and seed release timing. Several studies have demonstrated that electrically driven precision seed meters can significantly improve seed spacing accuracy compared with traditional mechanically driven systems^10,13^ . In addition, electric metering systems allow flexible adjustment of seed spacing and planting density through digital control, making them suitable for modern precision agriculture systems.

In parallel with these technological developments, agricultural robotics has gained considerable attention as a transformative approach for improving farming efficiency and sustainability. Remote-controlled agricultural robots are increasingly being developed to perform a variety of field operations, including seeding, weeding, spraying, harvesting, and decreasing operator exposure to repetitive, hazardous, or time-critical tasks. Robotic seeding systems utilize autonomous navigation and electrically controlled seed delivery mechanisms to improve planting precision and decrease operator dependency^6,14,15^. Autonomous weeding robots employing machine vision and artificial intelligence significantly reduce herbicide usage by providing site-specific weed removal and mechanical weed control^16–18^. Similarly, robotic spraying platforms equipped with sensor-based variable-rate application systems have enhanced pesticide application efficiency while reducing environmental contamination^19–21^. Robotic systems have been effectively used in harvesting operations for high-value crops such as vegetables and fruits, where robotic manipulation and computer vision enable selective harvesting under field and greenhouse conditions^22–25^. These developments show the growing role of robotics in agricultural mechanization, where remote-controlled machines are increasingly capable of performing complex field tasks with high precision and operational consistency. Despite these advancements, according to systematic reviews, harvesting and weeding robots are very mature, whereas seeding robots are largely underexplored, despite their strategic relevance in the agricultural production cycle, especially in open-field row-crop farming^8^. The integration of electric precision seed metering mechanisms with remotely controlled robotic platforms powered by renewable energy sources remains an active research area requiring further development and field validation.

Another important challenge facing modern agriculture is the need to reduce energy consumption and environmental impacts associated with agricultural machinery. Conventional farm equipment relies heavily on fossil fuels, which contribute to greenhouse gas emissions and increased production costs. Therefore, integrating renewable energy sources into agricultural machinery represents an important strategy for improving sustainability in crop production systems. Solar energy, in particular, offers a clean and renewable power source that can be effectively used to operate small remote-controlled agricultural machines. Solar-powered robotic platforms can perform field operations with minimal environmental impact while reducing dependence on fossil fuels^26^ .

Solar-powered agricultural robots represent a promising approach to sustainable automation, particularly in regions with high solar radiation and limited access to traditional fuel resources. Integrating photovoltaic energy systems with electric drive and control units enables agricultural robots to operate with lower operational costs and environmental impact. Several studies have confirmed the feasibility of solar-powered robotic platforms for agricultural monitoring and field operations^5,6,27^. However, the integration of solar-powered robotic systems with precision seed-metering mechanisms remains relatively limited in the current literature. In particular, ensuring accurate synchronization between the robot forward speed and seed release timing represents a key challenge in the design of robotic planting systems.

The development of an electrically powered seed-metering system integrated into a solar-powered remote-controlled robot offers the potential to improve planting precision while reducing energy use and dependence on fossil fuels. Electric seed metering mechanisms provide precise control over disc rotation, enabling accurate synchronization of robot travel speed and seed-release timing, which is essential for ensuring consistent seed spacing in precision planting systems^10^ . Reviews of field robots reveal a growing number of electrically driven platforms and note the potential of on-board photovoltaic (PV) energy harvesting to reduce dependence on fossil fuels and fixed charging infrastructure, particularly for long-duration, low-speed jobs^4,28^.

Although numerous studies have investigated precision planting technologies and electric seed-metering devices, limited research has focused on integrating these technologies into solar-powered remote-controlled robotic platforms. Furthermore, comprehensive evaluations of seed metering performance under varying operating conditions are required to assess the practical feasibility of such systems for precision agriculture applications.

Therefore, the objective of this study was to design, develop, and evaluate a solar-powered remote-controlled agricultural robot equipped with an electric seed metering device for precision seed planting. The system design, control strategy, and field performance were evaluated to determine the effectiveness of the proposed robotic planting platform under different operating conditions. The results of this research contribute to the development of sustainable robotic planting technologies for precision agriculture. In addition, the study aims to provide a reference architecture and performance benchmarks for next‑generation, multipurpose, solar-powered robotic platforms in precision agriculture.

## Materials and methods

### System overview

**Fig. 1:**
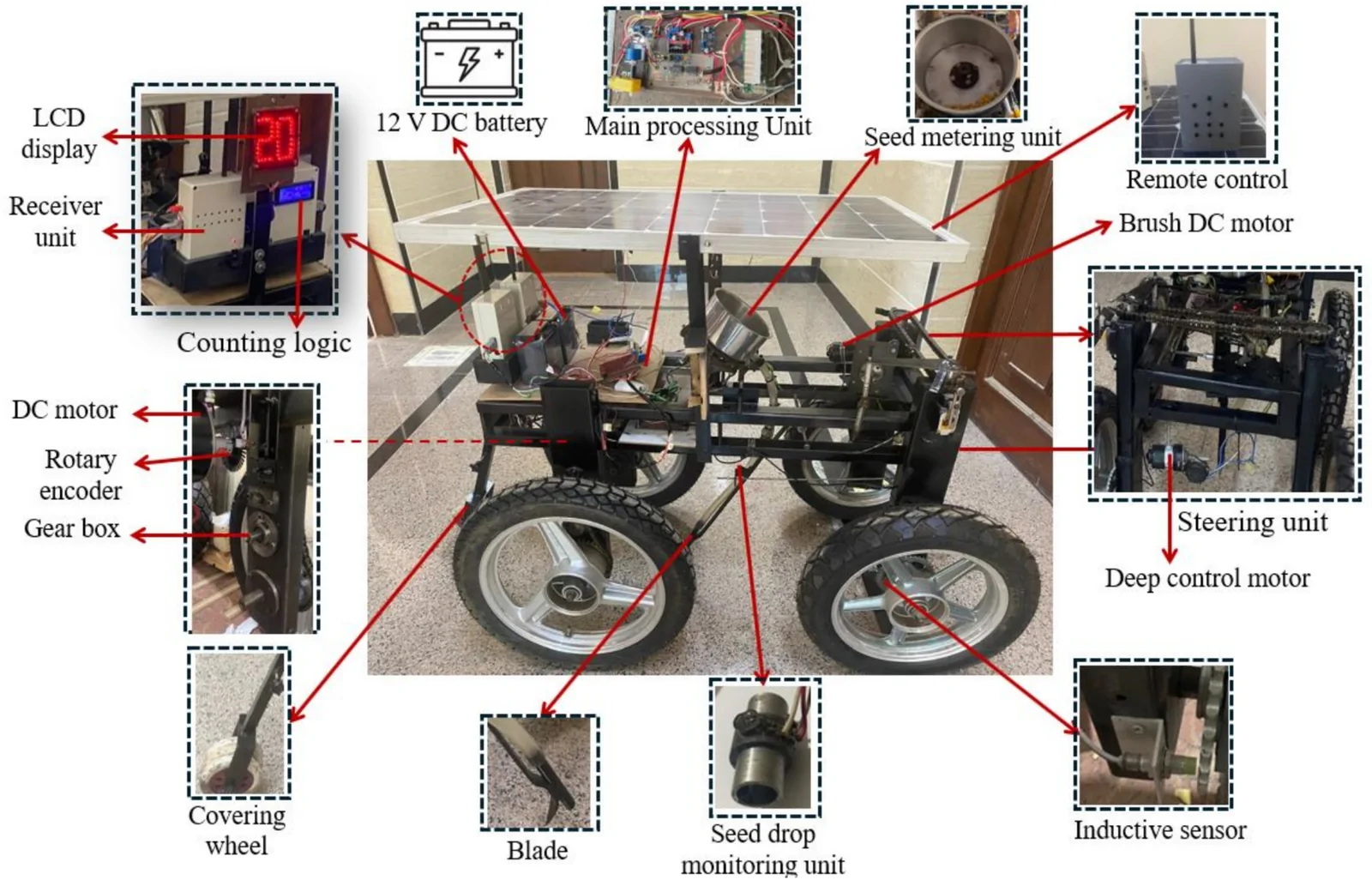
Rendering of the agricultural robot platform showing the major assemblies for precision seed planting.

The developed system is a solar-powered, remotely controlled agricultural robot designed for precision seed planting. The robot integrates a photovoltaic power supply, a battery storage system, an electric drive unit, an embedded controller, and an electrically driven seed-metering mechanism. The design focuses on achieving accurate seed placement while maintaining energy autonomy during field operation. The robot was composed of three primary subsystems: the mechanical subsystem, the electrical and power subsystem, and the control and sensing subsystem. The developed robot in this study can be used to seed various crop types, including corn, in both open-field and research experiments, as shown in Fig. 1.

**Fig. 2:**
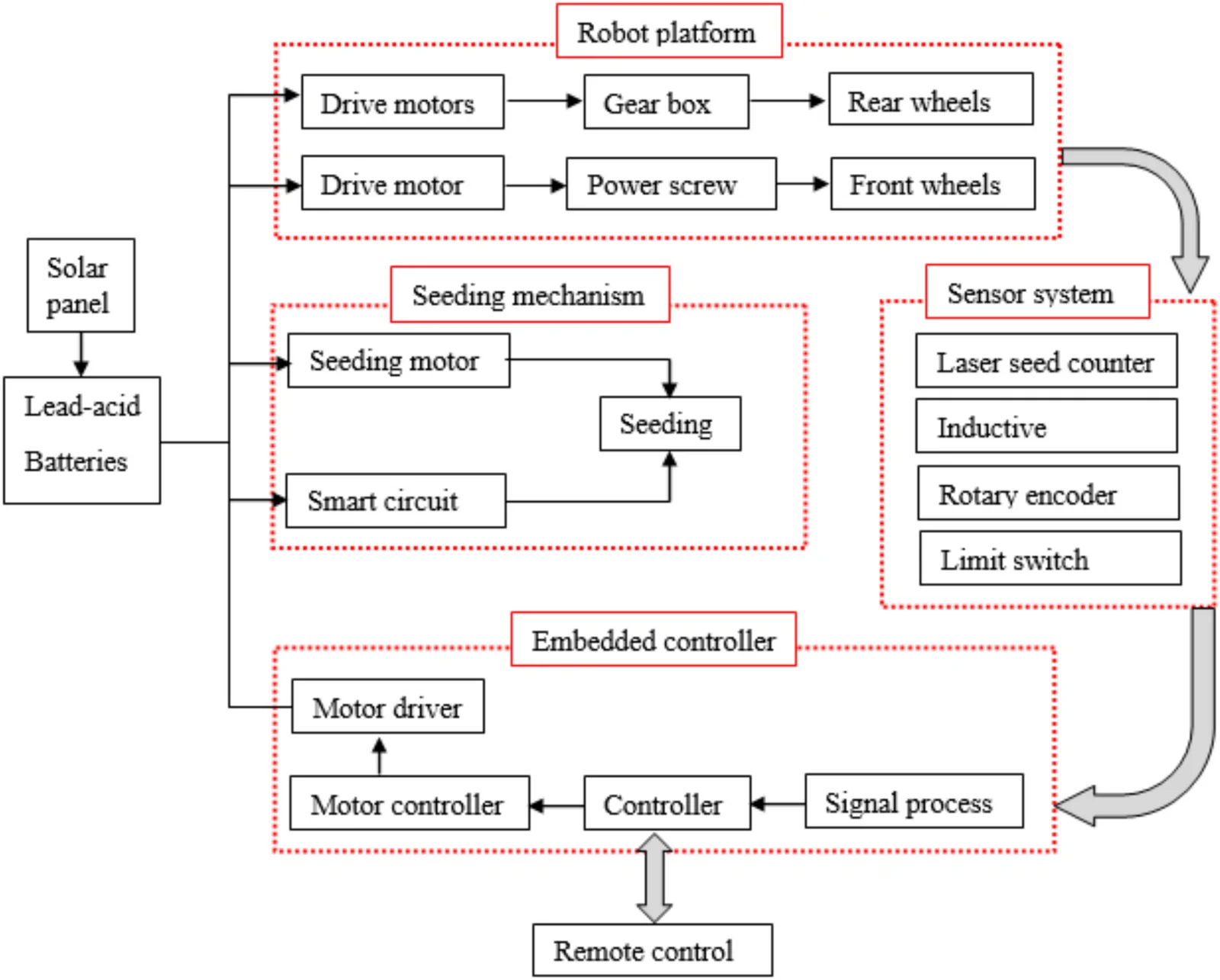
System block diagram of the robot.

The design, fabrication, and experimental evaluation of the solar powered wirelessly controlled robot were conducted in the Department of Agricultural Engineering, Faculty of Agriculture, Kafrelsheikh University, Egypt. Laboratory calibration tests were performed before field experimentation to ensure system stability and operational accuracy. The system block diagram of the corn precision seeding robot is illustrated in Fig. 2.

### Mechanical structure of the robot

**Fig. 3:**
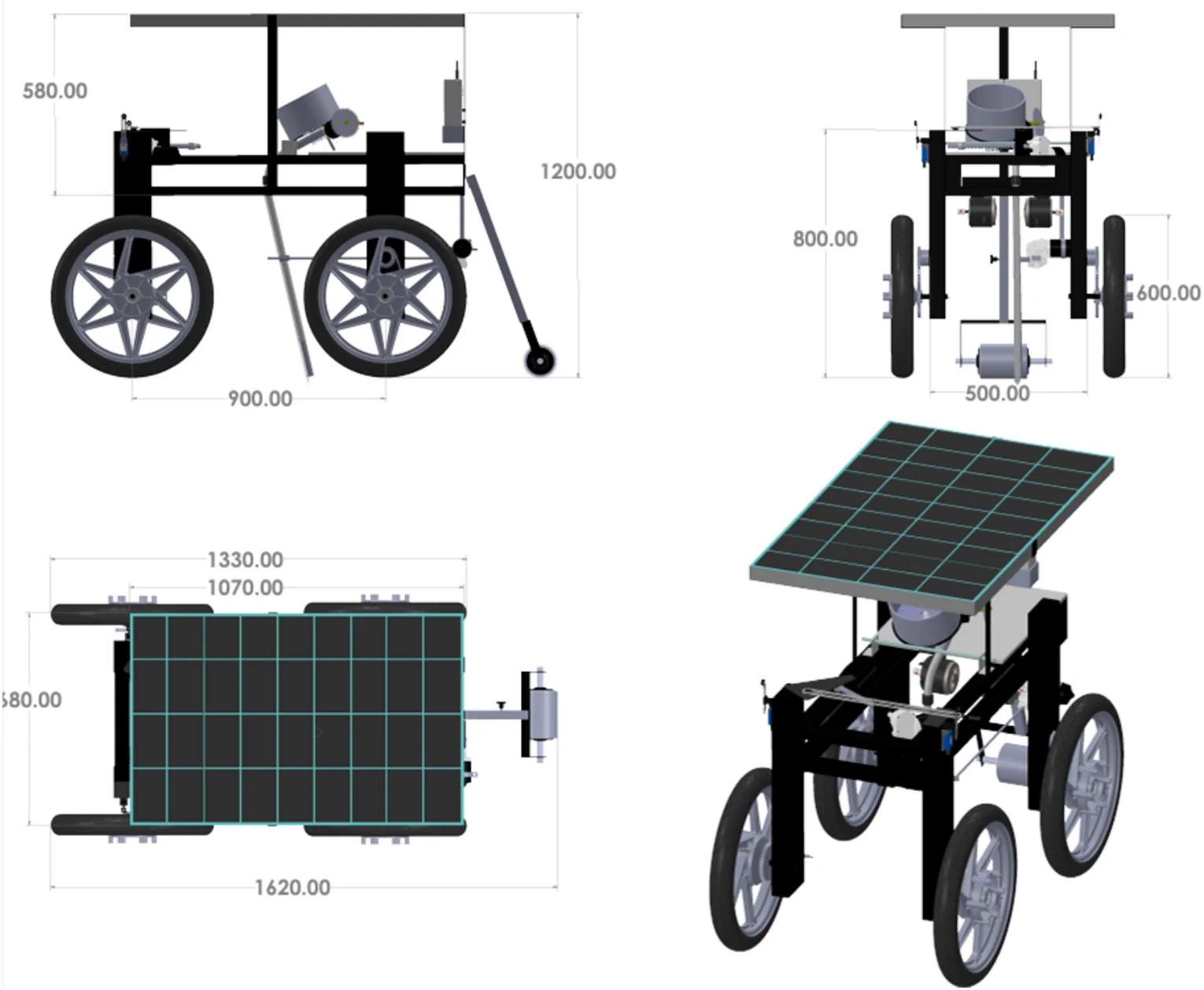
Three-dimensional CAD model of the solar-powered precision seed planting robot showing front, side, top, and isometric views of the complete mechanical structure.

The solar powered remotely controlled robot was designed in accordance with field operating conditions and agronomic requirements for precision maize seeding. A four-wheel drive configuration was adopted to enhance traction and field mobility, while both the drive and steering systems were powered by DC motors to ensure accurate motion control. The mechanical structure was developed using Autodesk Fusion 360 CAD software to optimize component layout and structural integrity before fabrication. Special emphasis was placed on chassis strength, as it serves as the primary load-bearing framework supporting all mechanical and electrical subsystems. The robot frame was constructed using a lightweight steel frame to ensure structural stability while minimizing energy consumption. The final chassis dimensions were 1620 mm × 680 mm × 1200 mm (length × width × height), with a total system weight of approximately 160 kg, ensuring adequate stability and durability under field conditions suitable for experimental evaluation, as shown in Fig. 3.

### Steering unit design

The steering unit was developed to provide precise left–right directional control of the agricultural robot through an electromechanical auto-guidance mechanism. The system comprised a 24 V, 3 A, 60 rpm brushed DC motor, a lead screw transmission, dual limit switches, and a relay-based H-bridge motor driver. Motor rotation was converted into linear displacement using a stainless-steel Acme-thread lead screw (300 mm length, 15 mm diameter, 10 mm pitch) coupled to a bronze-lined nut, ensuring high load capacity and positional stability. The linear motion was mechanically translated into angular deflection of the front wheels.

To guarantee synchronized steering, two 14-tooth sprockets mounted on the front wheel axles were interconnected via a roller chain, enforcing identical angular displacement and improving directional stability. Mechanical end stops were defined using two limit switches (MJ-7108) positioned at the screw travel boundaries, providing discrete feedback to prevent over-travel and enhance repeatability.

#### Steering kinematic and torque analysis

The steering system was analytically designed to determine the required motor power, screw torque, axial thrust force, steering torque at the wheel axis, and actuation time. Classical machine design equations were applied to ensure proper sizing of mechanical and electrical components. Standard procedures have been followed^29^.

*Motor power:* The motor output power was calculated from the electrical input parameters as:

1 $$\begin{array}{cccc}& P=VI{\eta}_{m}& & \end{array}$$

where *P* is the motor output power (W), *V* is the supply voltage (V), *I* is the current (A), and ${\eta}_{m}$ is the motor efficiency.

Substituting the rated values:

$$P=24\times 3\times 0.90=64.8\text{ W}$$

*Motor torque:* The torque developed by the motor was determined from the rotational power relationship:

2 $$\begin{array}{cccc}& P=T\omega & & \end{array}$$

The angular velocity is given by:

3 $$\begin{array}{cccc}& \omega =\frac{2\pi N}{60}& & \end{array}$$

where *T* is the torque (N·m), *ω * is the angular velocity (rad ${s}^{-1}$), and *N* is the rotational speed (rpm).

For $$N=60\mathrm{r}\mathrm{p}\mathrm{m}$$:

$$\omega =6.28 \, {\text{rad s}}^{-1}$$

$$T=\frac{P}{\omega }=\frac{64.8}{6.28}=10.32\text{ N}.m$$

Lead screw geometry: The mean diameter of the screw was calculated as:

4 $$\begin{array}{cccc}& {d}_{m}={d}_{o}-\frac{p}{2}& & \end{array}$$

where ${d}_{m}$ is the mean diameter (mm), ${d}_{o}$ is the major diameter (mm), and *p* is the pitch (mm).

$${d}_{m}=15-\frac{10}{2}=10\text{ mm}$$

*Screw efficiency:* The helix angle *α * was determined from:

5 $$\begin{array}{cccc}& \mathrm{t}\mathrm{a}\mathrm{n}\alpha =\frac{p}{\pi {d}_{m}}& & \end{array}$$

Assuming a friction coefficient *μ =0.1*, where:

$$\mu =\mathrm{t}\mathrm{a}\mathrm{n}\phi $$

The screw efficiency was calculated as:

6 $$\begin{array}{cccc}& {\eta}_{s}=\frac{\mathrm{t}\mathrm{a}\mathrm{n}\alpha (1-\mu \mathrm{t}\mathrm{a}\mathrm{n}\alpha )}{\mathrm{t}\mathrm{a}\mathrm{n}\alpha +\mu }& & \end{array}$$

The calculated efficiency was:

$${\eta}_{s}=0.737(73.7\mathrm{\%})$$

*Axial thrust force*: The axial thrust force generated by the lead screw was obtained from:

7 $$\begin{array}{cccc}& T=\frac{{F}_{a}L}{2\pi {\eta}_{s}}& & \end{array}$$

where ${F}_{a}$ is the axial thrust force (N), *L* is the screw lead (m), and ${\eta}_{s}$ is the screw efficiency.

Rearranging Eq. (7):

8 $$\begin{array}{cccc}& {F}_{a}=\frac{2\pi {\eta}_{s}T}{L}& & \end{array}$$

Substituting the known values:

$${F}_{a}=\mathrm{4,776}\text{ N}$$

*Steering torque at the wheel axis*: The steering torque acting on the front wheel axis was estimated using:

9 $$\begin{array}{cccc}& {T}_{w}={F}_{a}r\mathrm{s}\mathrm{i}\mathrm{n}\theta & & \end{array}$$

where ${T}_{w}$ is the steering torque (N·m), *r* is the steering arm radius (0.165 m), and *θ * is the steering angle.

For forward alignment ($\theta ={90}^{\circ }$):

$${T}_{w}=788.1\text{ N.m}$$

For maximum steering angle ($\theta ={45}^{\circ }$):

$${T}_{w}=557.3\text{ N.m}$$

Linear velocity and actuation time: The linear velocity of the screw was calculated as:

10 $$\begin{array}{cccc}& v=Np& & \end{array}$$

where *v* is the linear velocity (mm min^-1^), *N* is the rotational speed (rpm), and *p* is the pitch (mm).

$$v=600 \, {\text{mm min}}^{-1}=10 \, {\text{mm s}}^{-1}$$

The time required to achieve a displacement *d* was determined from:

11 $$\begin{array}{cccc}& t=\frac{d}{v}& & \end{array}$$

For *d=100* mm:

$$t=10\text{ s}$$

So, the Linear force is approximately 4776.5 N, and it takes about 10 seconds to move 0.1 meters with the given parameters.

#### Control components

**Fig. 4:**
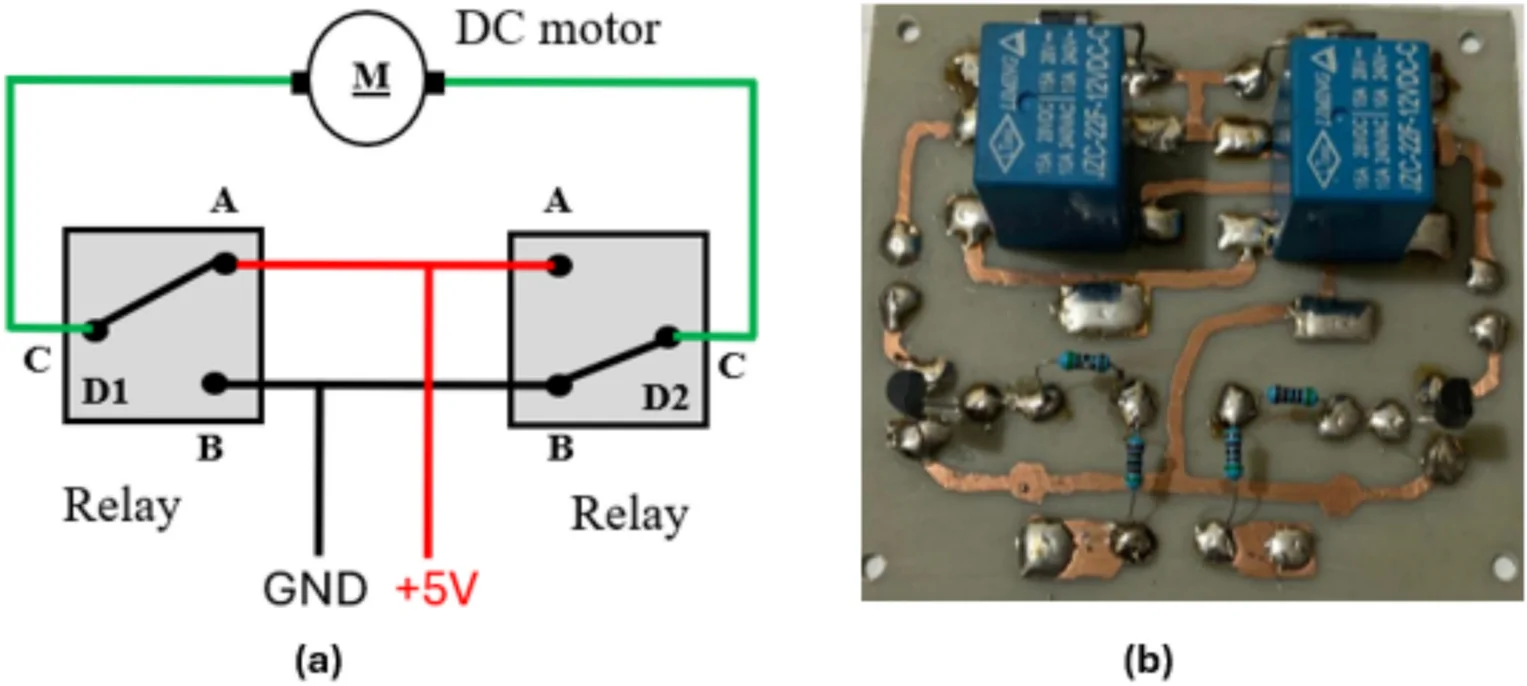
(**a**) Relay H-bridge circuit diagram, (**b**) Printed H-bridge circuit for steering unit.

Bidirectional motor control was achieved using two electromagnetic relays (JZC-22F-12VDC-C) configured in an H-bridge topology with transistor switching and flyback diode protection (Fig. 4). The driver enabled polarity reversal and neutral braking. The relay-based architecture provided electrical isolation, mechanical robustness, and reliable performance under agricultural field conditions.

### Drive unit and power transmission system

The drive unit was designed to convert electrical energy derived from solar power into controlled mechanical motion to propel the agricultural robot. The system consisted of two 250 W, 24 V brushed DC motors (MY1016; 2550–2650 rpm; 75–85% efficiency), powered by two 12 V batteries charged through photovoltaic panels mounted on the robot chassis. The motors were connected in parallel with a reversed polarity configuration to enable forward and backward motion.

Mechanical power was transmitted to the rear wheels through a locally manufactured spur-gear gearbox designed using CAD-based dimensional optimization. Based on a target no-load travel speed of 2.4 km h⁻1 and a rear wheel diameter of 0.6 m, the required wheel speed was calculated as 21.23 rpm. Considering a motor speed of 2550 rpm under load, the theoretical gear ratio was 120:1; however, a practical ratio of 117:1 was implemented. The resulting rear axle torque reached approximately 221–234 Nm, providing a tractive force of 737–780 N (≈75–80 ${\mathrm{k}\mathrm{g}}_{\mathrm{f}}$), sufficient for field operation.

**Fig. 5:**
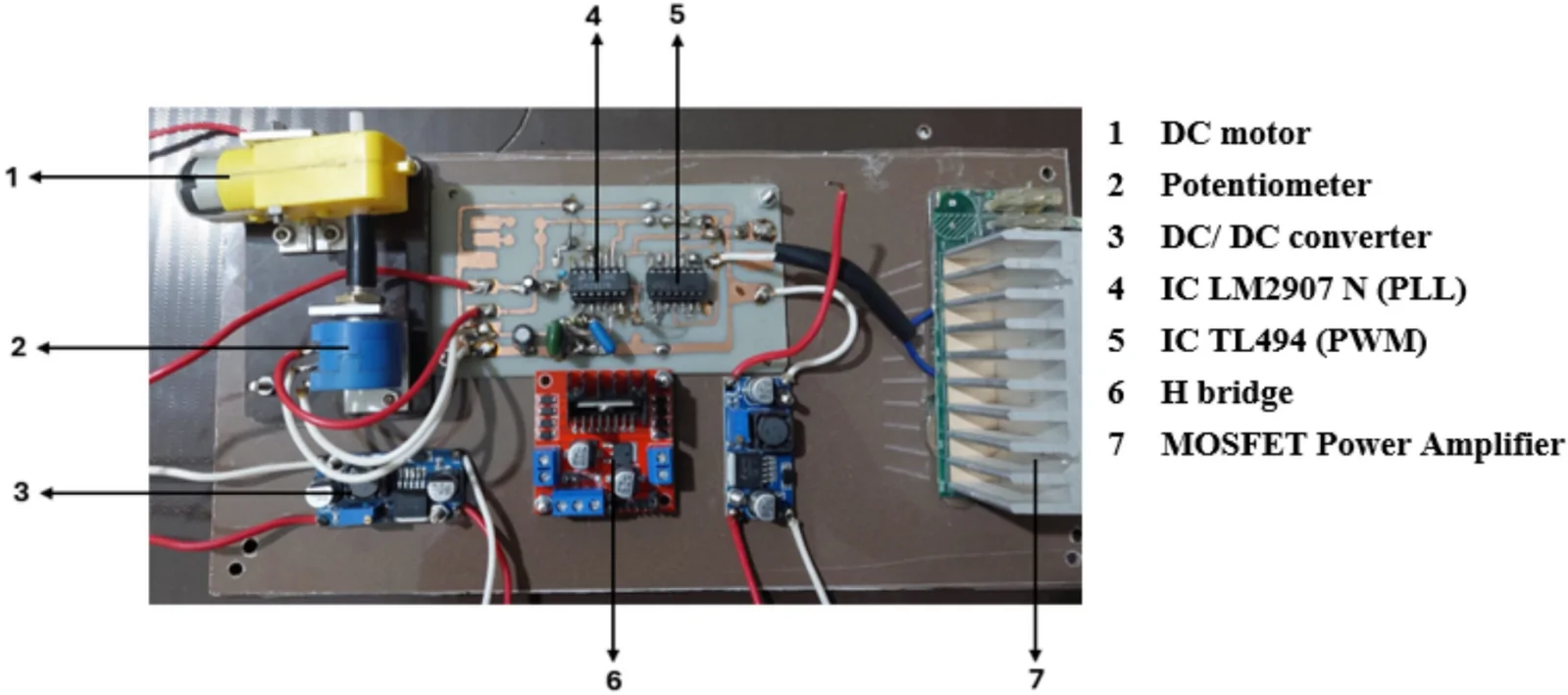
Electronic components of the phase-locked loop (PLL) and pulse-width modulation (PWM) circuits are integrated into the agricultural robot control system.

Bidirectional motion control was achieved using a dual high-current H-bridge (120 A, 24 V relays). Motor speed regulation was implemented through pulse-width modulation (PWM, TL494 IC), while rotational synchronization and closed-loop speed control were achieved using a phase-locked loop (PLL, LM2907N). A 50-pulse rotary encoder coupled with an optocoupler provided real-time feedback for speed correction. The integrated PLL–PWM architecture ensured stable lower-speed operation, higher torque, and enhanced traction efficiency under variable load conditions, as shown in Fig. 5.

### Electric seed metering system

#### System configuration and operating principle

**Fig. 6:**
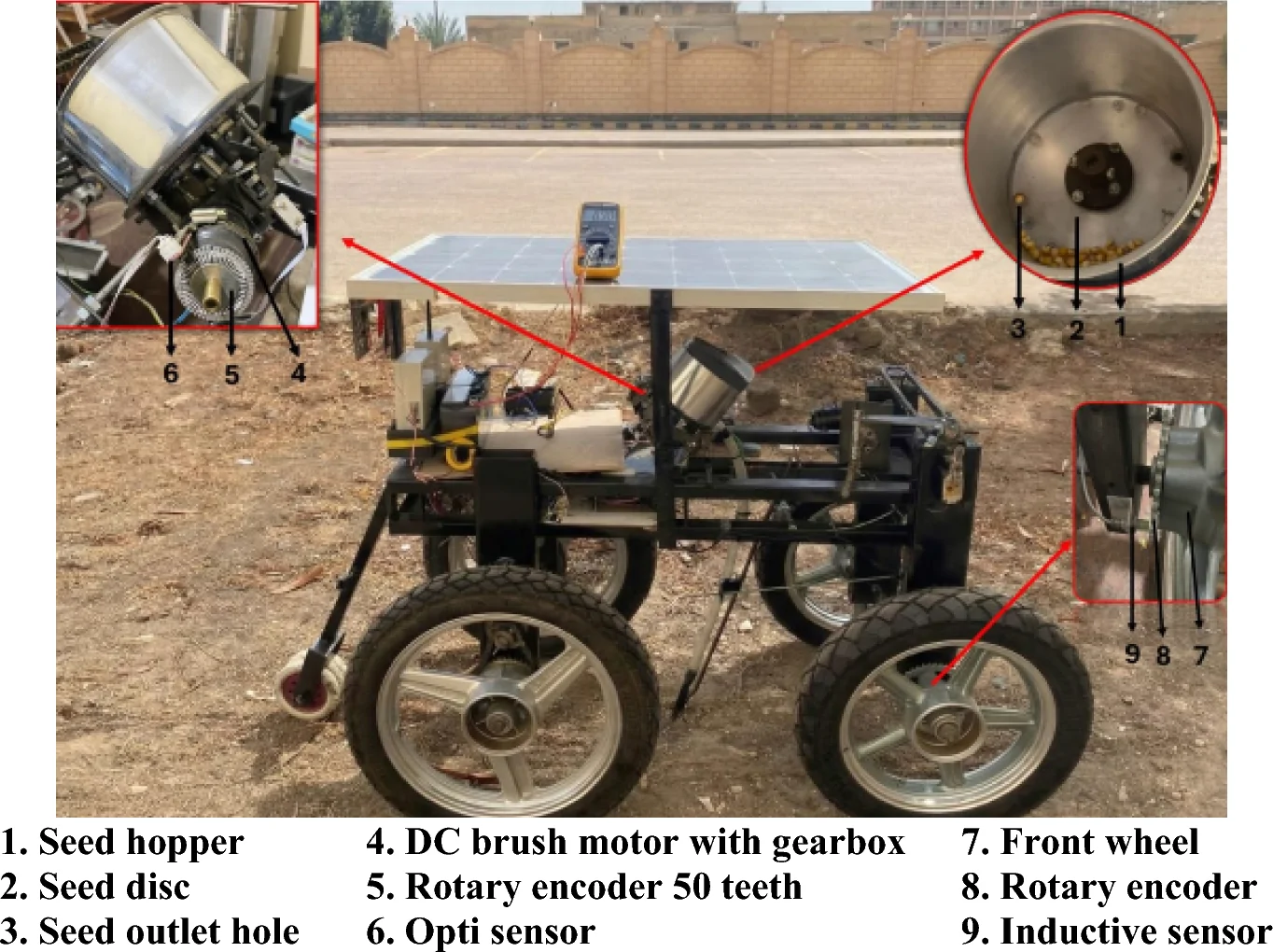
Structure configuration of the developed electric seed-metering device.

A precision electric seed metering unit was designed and integrated into the wirelessly controlled agricultural robot to achieve controlled seed delivery and uniform intra-row spacing. The metering system consisted of a seed hopper, rotating seed plate, DC geared motor, rotary encoders, inductive sensor, electronic control circuit, and a seed monitoring module. The structural configuration of the developed metering mechanism is shown in Fig. 6.

The operating principle relied on synchronizing the rotational motion of the seed metering disc with the forward movement of the robot via a closed-loop control strategy. A rotary encoder mounted on the robot front wheel generated pulse signals proportional to the travelled distance. These pulses were processed by the control system and used as a reference signal for regulating the rotational speed of the metering motor.

A phase-locked loop (PLL)–based control approach was implemented to maintain synchronization between the motor rotation and the reference signal. Each encoder pulse triggered a controlled angular displacement of the seed disc corresponding to one-eighth of a rotation, equal to the number of seed holes on the disc. Once the required displacement was achieved, an optical feedback sensor mounted on the motor shaft generated a stop signal to terminate motor rotation.

This feedback mechanism allowed the system to compensate for speed variations and ensured accurate seed release timing. Consequently, the developed electric metering system maintained consistent seed spacing by dynamically adjusting motor speed according to the robot travel velocity.

#### Mechanical design of the metering unit

The mechanical assembly consisted of a seed hopper, seed metering disc, mounting frame, and seed delivery tube. The hopper was fabricated from aluminum with a cylindrical geometry (18 cm diameter) to ensure a continuous seed supply to the metering device. Seeds were transferred from the hopper to a rotating seed disc manufactured from polycarbonate with a diameter of 17.6 cm and a thickness of 0.6 cm.

The seed disc contained eight equally spaced holes designed according to the size characteristics of the maize variety used in the experiment (Hybrid 368) to ensure single-seed pickup. The disc rotation was driven by a 24-V geared DC motor (60 rpm) that provided controlled rotational motion. The mechanical components were mounted on a galvanized iron frame integrated with the robot chassis, which also supported the motion-sensing encoder.

#### Sensing and control hardware

The sensing and control architecture included motion sensors, motor control modules, and an embedded processing unit. An Arduino Uno microcontroller based on the ATmega328P processor served as the central control unit responsible for signal acquisition, motor control, and system monitoring.

**Fig. 7:**
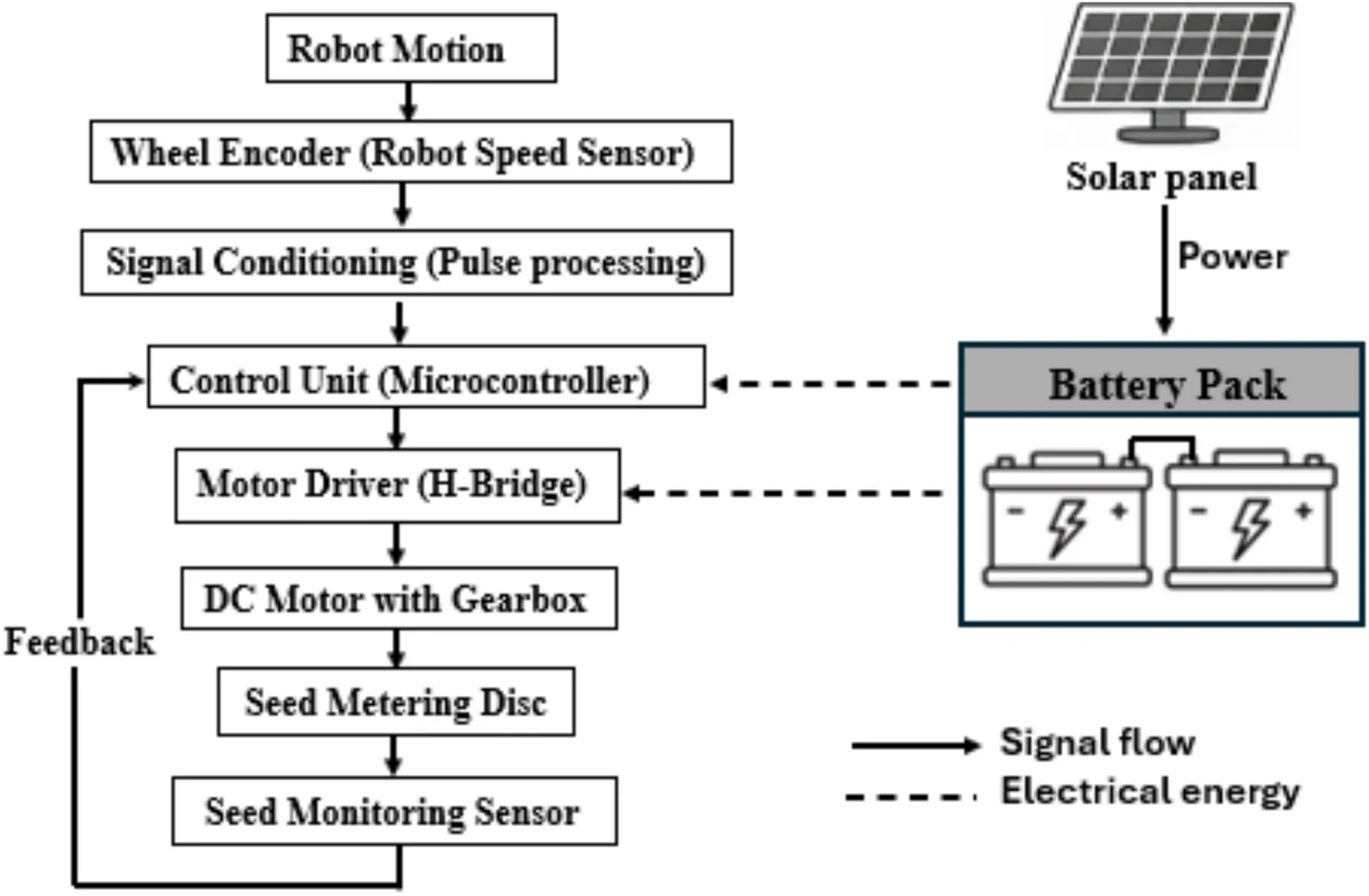
Simplified block diagram of the control architecture for the electric seed-metering system of the precision planting robot.

Motor actuation was achieved using an L298N dual H-bridge motor driver, which regulated the rotational speed of the DC motor driving the seed metering disc. Three rotary encoders were employed to provide motion feedback and synchronization, as shown in Fig. 7.

The first encoder (38 teeth) was mounted on the robot front wheel to measure forward displacement and generate reference pulses corresponding to target planting spacings of 10, 15, 20, and 25 cm. The second encoder (50 teeth) was installed on the motor shaft and provided feedback for PLL-based speed synchronization. A third encoder with eight divisions was aligned with the seed disc holes to trigger seed release events.

An inductive proximity sensor was positioned adjacent to the wheel encoder to detect each tooth passage and generate square-wave pulses representing robot motion. These signals were transmitted to the microcontroller to regulate motor speed and maintain the desired seed spacing.

Robot speed was determined using a frequency-to-voltage conversion circuit based on an LM2907 integrated circuit, which converted the encoder pulse frequency into an analog voltage signal. This signal was read by the microcontroller and used to generate a corresponding pulse width modulation (PWM) control signal for motor speed regulation.

#### Seed monitoring and counting system

A seed monitoring system was developed to detect and record individual seed delivery events. The sensing mechanism used an infrared laser emitter and receiver installed across the seed tube. When a seed passed through the sensing zone, it interrupted the light beam, producing a signal detected by the receiver. The signal was processed using an LM393 comparator to remove noise and generate a clean digital pulse representing a seed drop. Each pulse was transmitted to the microcontroller for counting and monitoring. The electronic components were powered using a regulated 5-V supply provided by an LM7805 voltage regulator.

The processed data were displayed in real time using an LCD module connected to the microcontroller. The display provided operational information, including seed count detected by the monitoring sensor, theoretical seed count derived from encoder pulses, planting distance, seed rate, robot speed, and error values between expected and actual seed delivery.

#### Embedded control algorithm

The metering system operated using a closed-loop control algorithm implemented in the Arduino IDE environment and executed on the ATmega328P microcontroller. The algorithm continuously processed encoder signals to estimate robot speed and displacement. Based on the measured speed, the controller generated PWM signals to regulate the rotational speed of the seed metering motor through the motor driver module. This ensured synchronization between the robot travel velocity and the seed disc rotation.

At the maximum robot operating speed of 2.5 km h⁻1 (0.69 m s⁻1), the expected pulse frequency from the wheel encoder was calculated using Eq. (12):

12 $$N=\frac{V}{C}\times T$$

where *N* is the number of pulses per second, *V* is the robot forward speed (m s⁻1), *C* is the wheel perimeter (m), and *T* is the number of encoder teeth.

For the experimental configuration (wheel perimeter = 1.77 m and encoder teeth = 38), the calculated pulse frequency was approximately 14.8 pulses s⁻1. The coordinated interaction between motion sensing, motor control, and seed detection allowed the system to maintain consistent seed spacing and reliable planting performance under different operating conditions.

### Wireless remote-control system

A wireless remote-control system was integrated into the solar-powered agricultural robot to enable real-time control of operational parameters and facilitate safe field operation. The system consisted of a handheld transmitter unit and an onboard receiver module interfaced with the robot control unit. Communication between the two units was established using a 2.4 GHz radio frequency (RF) link based on the NRF24L01 transceiver module, which provided reliable long-range communication with low power consumption and minimal interference under open-field conditions.

The transmitter unit was designed to generate coded digital commands corresponding to various robot functions. These signals were received and decoded by the onboard receiver module and subsequently processed by the microcontroller to control the robot actuators. Through this wireless interface, the operator was able to adjust several operational parameters during planting operations, including robot forward speed, seed metering disc rotational speed, seed spacing, and seed depth. Directional navigation commands such as forward, backward, left, and right movements were also transmitted through the remote controller and executed through motor driver circuits connected to the wheel drive motors.

#### Transmitter unit

**Fig. 8:**
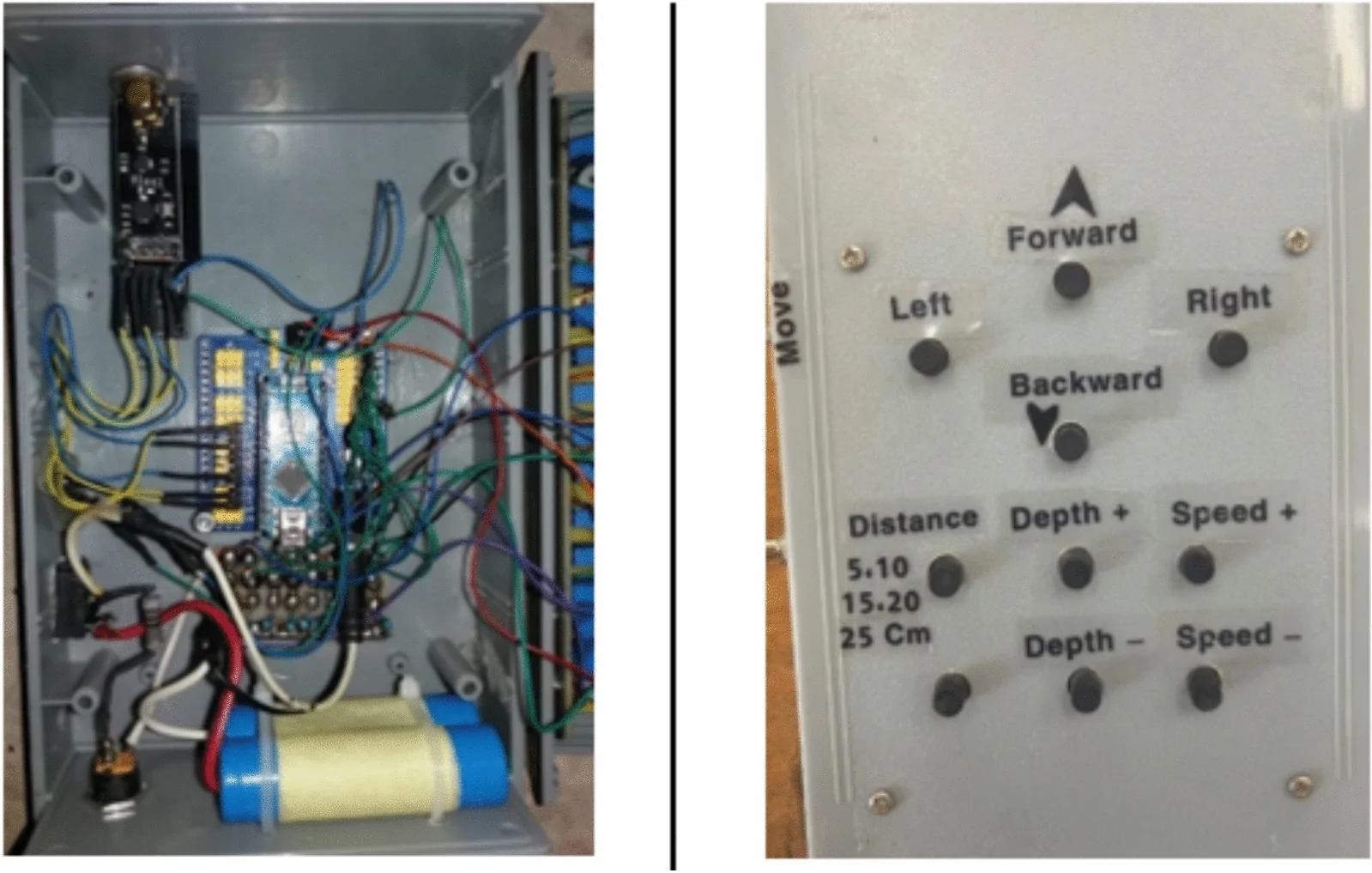
The internal electronic components and the control push-button interface of the wireless remote controller are used for precision seed planting.

The transmitter unit consisted of a handheld controller integrating an Arduino Nano microcontroller and an NRF24L01 RF communication module operating at 2.4 GHz. The control interface included push buttons and joysticks that enabled the operator to adjust robot travel speed, seed metering disc rotation, seed spacing, and seed depth in real time. Directional commands for robot navigation were also provided through the control interface. The internal electronic components and control interface of the transmitter are illustrated in Fig. 8.

#### Receiver unit

**Fig. 9:**
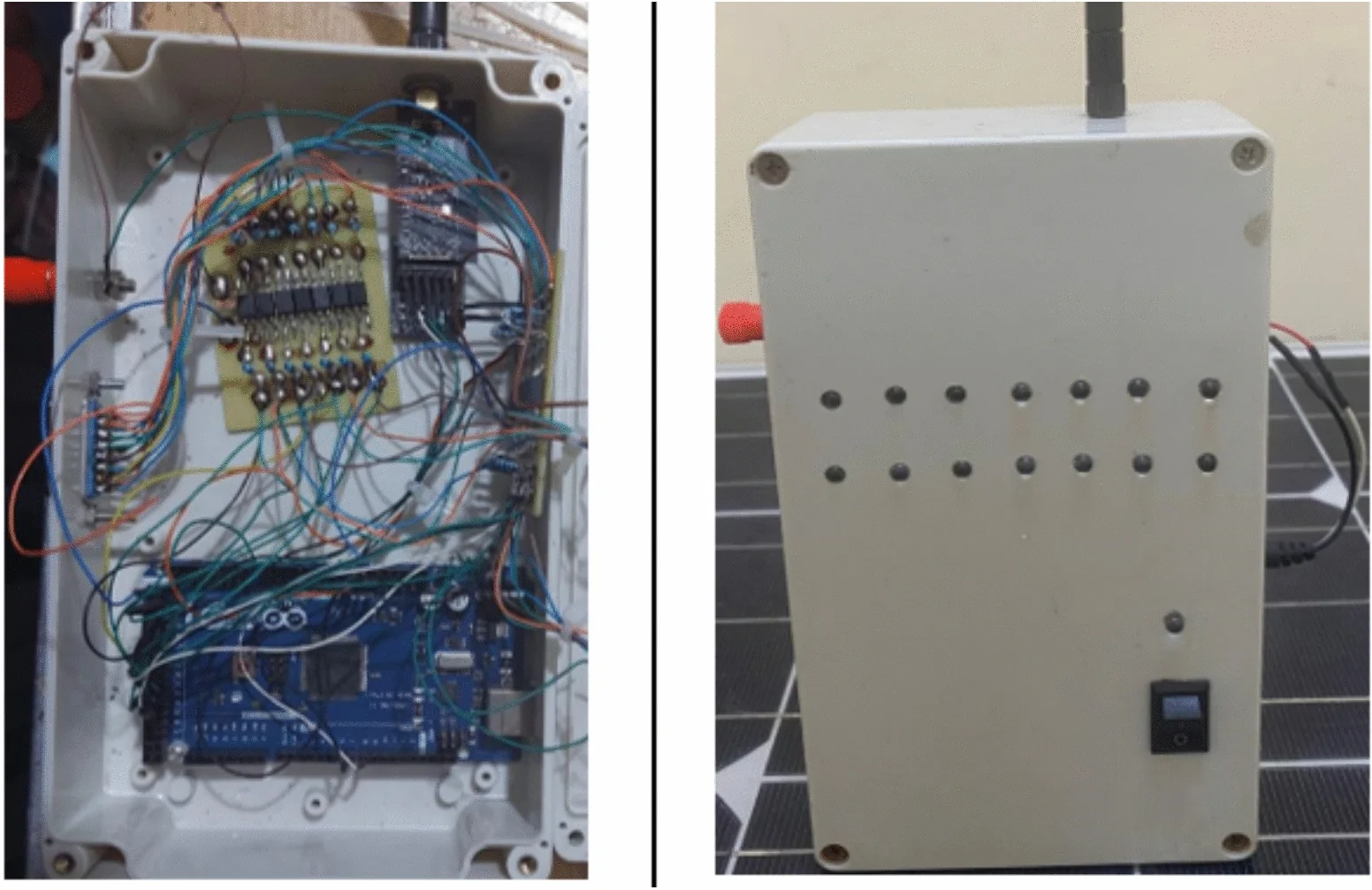
The receiver unit components from inside and the command LEDs in the receiver unit.

The receiver unit was installed on the robot chassis and comprised an NRF24L01 RF receiver module interfaced with an Arduino Mega microcontroller. The module received and decoded the transmitted digital signals and relayed them to the control algorithm programmed in the microcontroller. The decoded commands were then converted into electrical control signals for the corresponding actuators, including the wheel drive motors for robot navigation, the seed metering motor for regulating disc rotation, and the actuator responsible for seed depth adjustment. The receiver unit configuration is shown in Fig. 9.

#### Communication and control software

**Fig. 10:**
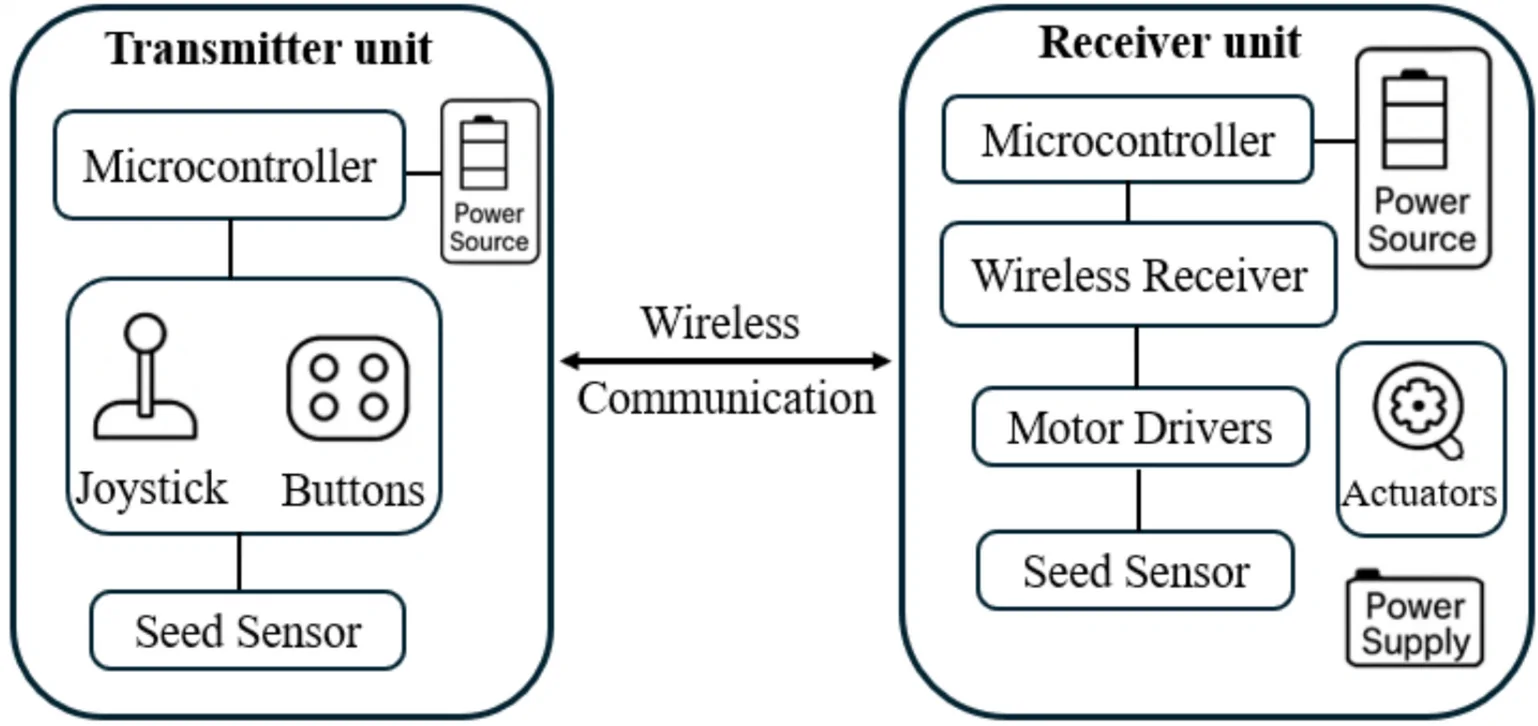
Block diagram of a wireless remote-control system for an agricultural robot.

The control software enabled real-time wireless communication with an effective range of approximately 1200 m under open-field conditions. An LM35 temperature sensor was integrated into the system to monitor the operating temperature of the electronic components and motors. The sensor output was continuously read by the microcontroller and compared with predefined threshold values. When the temperature exceeded the allowable limit, the controller automatically reduced motor speed or stopped the system to prevent overheating. The overall architecture of the wireless control system and the Arduino control code are presented in Fig. 10.

### Seed depth adjustment unit

A seed depth adjustment mechanism was developed to control the vertical position of the seed opener during planting operations. The unit consisted of a DC brush motor coupled with a lead screw mechanism that converted rotary motion into controlled linear displacement. The motor operated at a nominal speed of 3600 rpm and was connected to a 1:60 reduction gearbox to ensure precise and stable movement. The gearbox output was mechanically coupled to a lead screw with a diameter of 6 mm and a pitch of 1 mm, enabling accurate vertical adjustment of the seed opener.

**Fig. 11:**
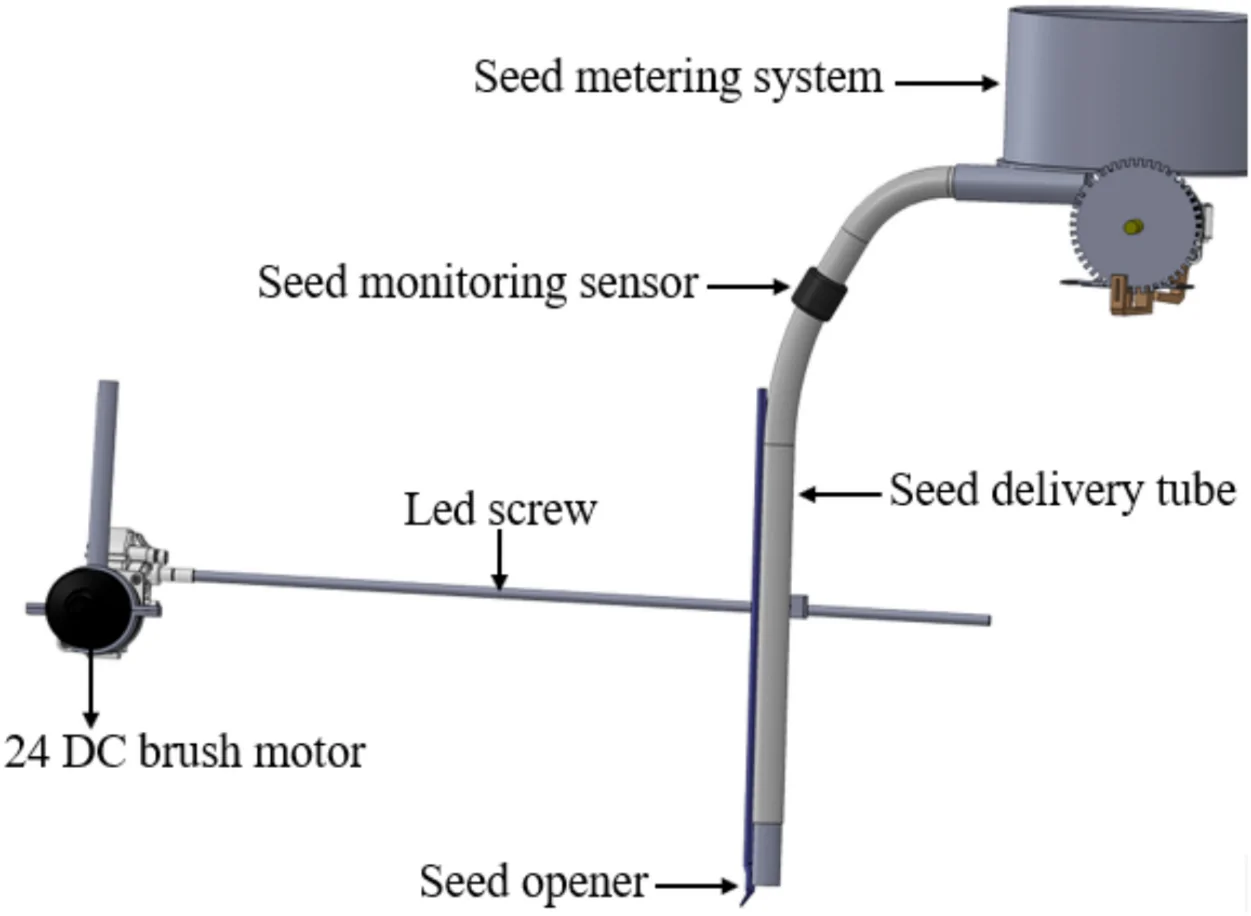
The seed depth adjustment unit.

The control system included an Arduino Uno R3 microcontroller, a dual H-bridge motor driver, and a 24 V DC power supply derived from the robot battery. A regulated 5 V output powered the microcontroller and peripheral electronic components. The motor rotation was controlled through wireless commands, allowing the lead screw to rotate clockwise or counterclockwise, which resulted in upward or downward movement of the seed opener, as shown in Fig. 11. This mechanism enabled real-time adjustment of the seed penetration depth to accommodate different soil conditions and crop requirements.

### Power source

**Fig. 12:**
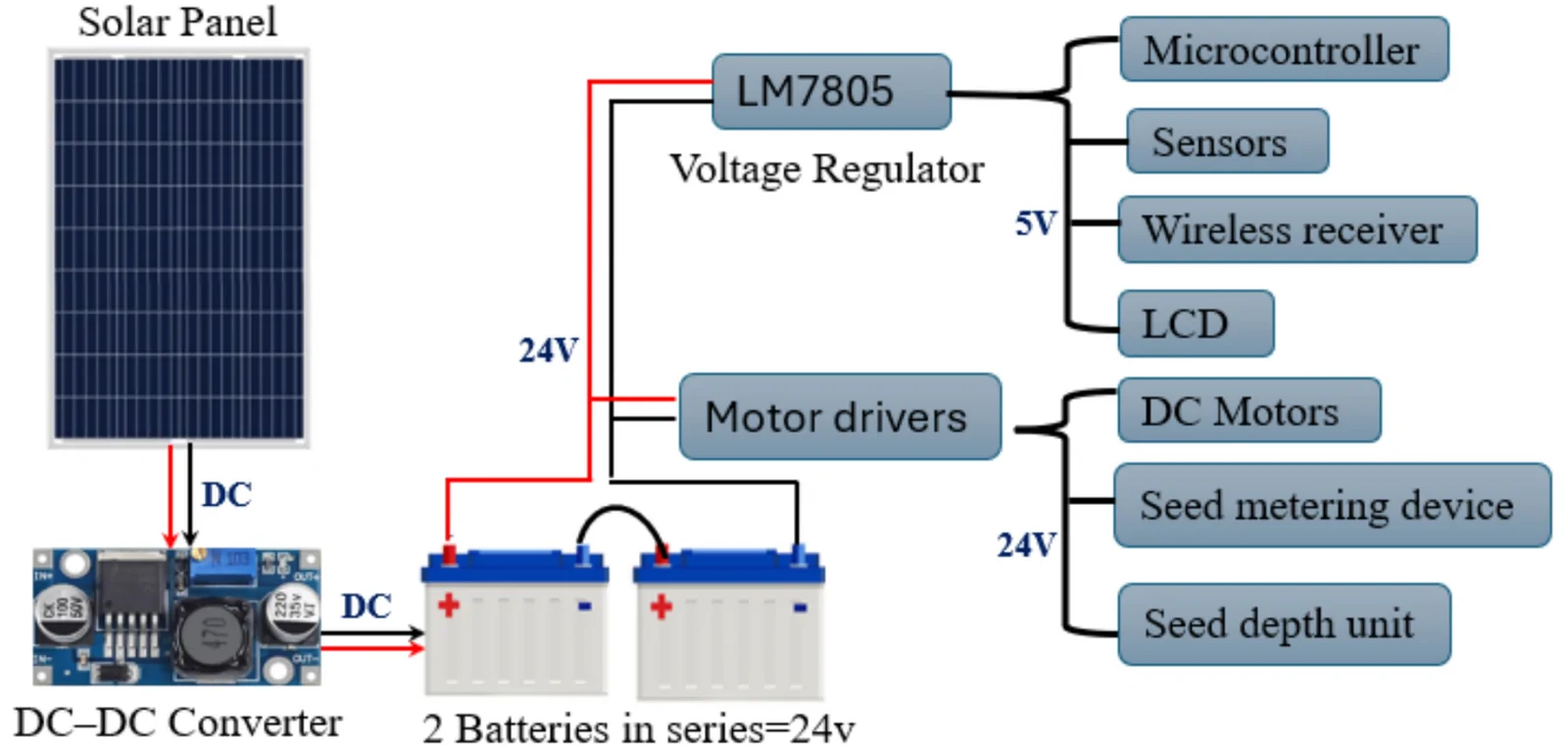
Power source block diagram of solar-powered agricultural robot for precision seed planting.

The agricultural robot was powered by a hybrid solar–battery energy system designed to provide stable and continuous power during field operations. A 100 W monocrystalline photovoltaic (PV) panel was mounted on the top frame of the robot to maximize solar energy capture. The solar output was regulated using an LM2596 DC–DC buck converter (3–40 V input, 1.5–35 V adjustable output) to ensure safe and stable charging of the battery system. The energy storage unit consisted of two 12 V, 8 Ah sealed lead-acid batteries connected in series to provide a 24 V DC supply for the robot (Fig. 12).

The stored electrical energy of the battery system was calculated as:

13 $${E}_{battery}=V\times Ah=24\times 8=192\text{ Wh}$$

**Fig. 13:**
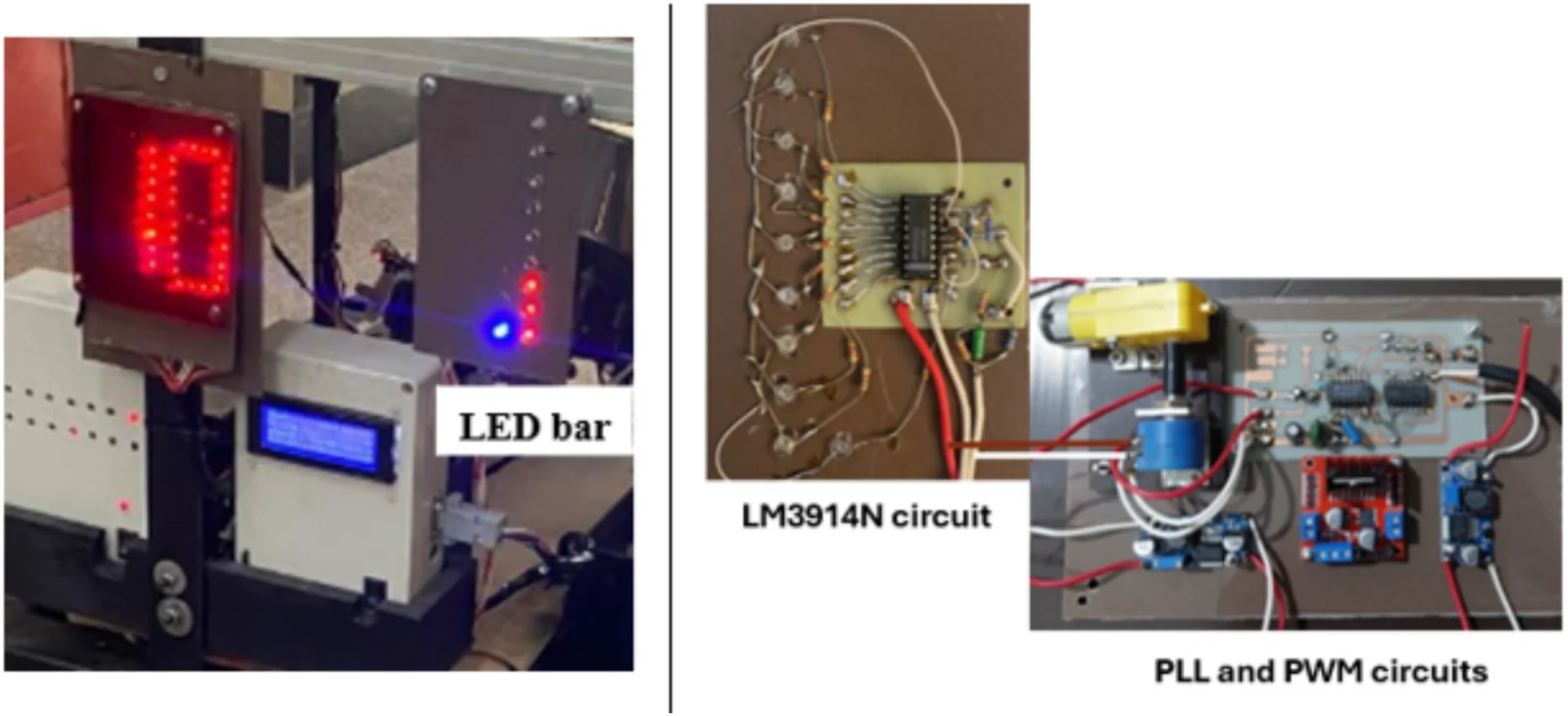
Voltage-to-speed indicator circuit based on the LM3914N integrated circuit for forward speed calibration of the agricultural robot.

The batteries supplied power to the traction motors, seed metering unit, seed depth adjustment mechanism, and control electronics. A voltage regulator (LM7805) provided a stable 5 V supply for the microcontroller, sensors, and wireless communication modules, while the 24 V output was used to drive the DC motors through motor driver circuits (Fig. 13).

Power consumption of the robot was experimentally determined by measuring the operating current at different forward speeds using a digital ammeter. A constant current of 0.25 A, required for electronic components such as the control unit and display modules, was subtracted from the measured values to obtain the net mechanical power consumption. The electrical power demand was calculated as:

14 *P=V× I*

For estimating the photovoltaic contribution, an effective photovoltaic output of 80 W (80% of the rated panel power) was assumed under typical operating conditions. This empirical estimate was used solely as a baseline for calculating the Solar Power Supply Ratio (SPSR) as an energy balance indicator. It was not intended to represent experimentally measured photovoltaic conversion efficiency or irradiance-based PV performance.

15 $${P}_{solar,eff}=100\times 0.80=80\text{ W}$$

Battery-only operating time and solar energy utilization efficiency were subsequently calculated based on the measured robot power consumption.

Solar Power Supply Ratio (SPSR) was used to assess the ability of the photovoltaic system to meet the robot’s power demand and was calculated as:

16 $$SPSR(\%)=\frac{{P}_{PV,available}}{{P}_{robot}}\times 100$$

where ${P}_{\mathrm{P}\mathrm{V},\mathrm{a}\mathrm{v}\mathrm{a}\mathrm{i}\mathrm{l}\mathrm{a}\mathrm{b}\mathrm{l}\mathrm{e}}$ is the available photovoltaic power (W) and ${P}_{\mathrm{r}\mathrm{o}\mathrm{b}\mathrm{o}\mathrm{t}}$ is the measured robot power consumption (W). The SPSR indicates the proportion of the robot’s power demand supplied by the photovoltaic system and represents an energy-balance indicator.

### Physical properties, main parameters, and experimental methodology

#### Physical properties of maize seeds

Maize seeds from a commonly cultivated variety (Hybrid 368) were used to determine the design and operational parameters of the electric seed-metering device. The principal seed dimensions—length, width, and thickness—were measured using a digital Vernier caliper with a resolution of 0.01 mm, while the thousand-seed mass was determined using an analytical balance with 0.001 g accuracy. The average seed dimensions were 8.89, 8.28, and 5.49 mm for length, width, and thickness, respectively, with a thousand-seed mass of 290.06 g.

#### Main parameters and experimental measurements

To evaluate the performance of the solar-powered planting robot, several operational parameters were measured during laboratory and field experiments. Robot travel speed was determined by recording the time required to cover a 30 m distance, with test speeds of 0.42, 0.82, 1.2, and 1.6 km h⁻1. The rotational speed of the seed-metering disc was synchronized with the robot speed using encoder feedback and microcontroller control. Seed spacing was measured along a 30 m row after planting to assess distribution uniformity at target spacings of 10, 15, 20, and 25 cm. Battery performance was evaluated by monitoring the discharge voltage of the 24 V battery pack during continuous operation. The test was terminated when the pack voltage reached 23.0 V, corresponding to 11.5 V per individual 12 V sealed lead-acid battery, in accordance with the recommended discharge limit.

#### Experimental design and performance evaluation criteria

The field performance of the electric seed metering device integrated into the agricultural robot was evaluated using a factorial experimental design. Based on preliminary tests and design considerations, four seed disc inclination angles (30°, 35°, 40°, and 45°) were evaluated. The 35° inclination relative to the horizontal plane provided the best seed pickup and delivery performance and was therefore selected for the field experiments and performance evaluation. Four levels of robot forward speed (0.42, 0.82, 1.20, and 1.60 km h⁻1) and four levels of target intra-row seed spacing (10, 15, 20, and 25 cm) were tested. These ranges were selected based on preliminary experiments and common operating conditions for precision planting. Experimental data were analyzed using analysis of variance (ANOVA) to evaluate the effects of operating parameters on planting performance.

Seed placement quality was assessed using standard performance indices widely applied in agricultural machine evaluation, including the precision index (PI), miss index (MI), multiple index (MulI), and seed rate (SR). Metering performance was evaluated using ISO-based seed distribution indices^30,31^.

#### Calibration and performance evaluation of the robot forward speed control system

The forward speed of the agricultural robot was controlled using an electrical feedback system composed of a phase-locked loop (PLL) and pulse-width modulation (PWM) circuits, a signal conditioning unit, and a voltage-to-speed visual indicator based on the LM3914N integrated circuit. The analog voltage signal generated by the control system was processed through the LM3914N display driver, which converted the voltage levels into discrete LED bar indications, allowing real-time monitoring of robot speed, as shown in Fig. 13.

The LED display was mounted on the robot frame, where each illuminated LED represented a specific voltage and corresponding speed interval. The system provided eight discrete speed levels. Calibration was performed by measuring the time required for the robot to travel a known distance at each LED level, and the measured speeds were correlated with the corresponding voltage values to establish a voltage–speed relationship.

## Results and discussion

This section presents the experimental evaluation of the developed solar-powered remote-controlled agricultural robot equipped with an electric seed metering unit. The analysis focuses on the influence of robot forward speed and target seed spacing on seed placement performance and metering stability. System performance was evaluated using internationally accepted planter performance indicators, including precision index, miss index, multiple index, and seed rate, following the testing procedures specified in ISO 7256-1 and ISO 7256-2^30,31^.

### Optimization of seed-disc inclination angle

Preliminary experiments were conducted to determine the optimum seed-disc inclination angle under a robot forward speed of 1.2 km h⁻1 and a target seed spacing of 20 cm. Four inclination angles (30°, 35°, 40°, and 45°) were evaluated based on mean seed spacing, standard deviation, and coefficient of variation (Table 1). The 35° inclination angle produced the mean seed spacing closest to the target value (19.75 cm) and the lowest coefficient of variation (5.6%), indicating the best seed placement uniformity. One-way ANOVA confirmed that disc inclination significantly affected seed spacing (F = 7.14, p < 0.01). Therefore, the 35° inclination angle was selected for all subsequent experiments

**Table 1:** Effect of seed-disc inclination angle on seed spacing characteristics.

Seed disc inclination angle (°)	Mean seed spacing (cm)	Standard deviation (cm)	CV (%)
30	18.6	2.4	12.9
35	19.75	1.1	5.6
40	20.9	1.9	9.4
45	21.8	2.7	12.4

### Calibration and performance of the robot forward speed

The calibration results demonstrated a consistent and repeatable relationship between the applied motor voltage and the forward speed of the agricultural robot. Accurate speed calibration was essential because robot travel speed directly affects seed spacing and overall planting precision.

The voltage-to-speed monitoring system based on the LM3914N LED bar indicator provided a simple and reliable visual representation of speed variation. Each illuminated LED corresponded to a specific voltage range, allowing the operator to easily monitor and adjust the robot speed during field operation.

Experimental results showed a strong linear relationship between the applied motor voltage and robot forward speed within the operating range of 4–7 V. The corresponding measured speeds were 0.42, 0.82, 1.20, and 1.60 km h⁻1. Regression analysis confirmed this proportional relationship, expressed as:

$${V}_{r}=0.39V-1.14$$

where ${V}_{r}$ represents the robot forward speed (km h⁻1) and *V* is the applied motor voltage (V). The coefficient of determination (R2 > 0.98) indicates excellent linearity between voltage input and speed output. The calibrated speed range enabled flexible operation under different planting conditions. Lower speeds (0.42 km h⁻1) supported precise seed placement at small intra-row spacings, while higher speeds (up to 1.60 km h⁻1) allowed increased field capacity for larger spacing requirements. Moreover, the established voltage–speed relationship enabled the control unit to synchronize the seed metering disc rotation with the robot travel speed, ensuring consistent seed spacing during planting operations.

### Operating feasibility of the seed metering system under different speed–spacing conditions.

A feasibility analysis was conducted to determine whether the electric seed-metering mechanism could provide sufficient dwell time for reliable seed discharge under different combinations of robot forward speed and intra-row seed spacing. The analysis compared the available disc rotation time per seed with the minimum dwell time requirement of 0.2 s necessary for proper seed release.

The results indicated that the feasibility of the metering process was strongly influenced by robot travel speed. At the lowest speed (0.42 km h⁻1), all spacing levels (10–25 cm) provided large rotation time margins ranging from 0.66 to 1.94 s. These values greatly exceeded the minimum dwell-time requirement, ensuring stable seed release and highly reliable metering performance.

As the robot speed increased to 0.82 and 1.20 km h⁻1, the available release time decreased but still remained above the minimum threshold, maintaining feasible operating conditions. However, at the highest tested speed (1.60 km h⁻1), the available time margin became significantly smaller, particularly at the smallest seed spacing (10 cm), where the rotation time approached a near-critical value. Under these conditions, the metering system becomes more sensitive to disturbances such as vibration or soil irregularities, which may increase the probability of missed or delayed seed drops.

**Fig. 14:**
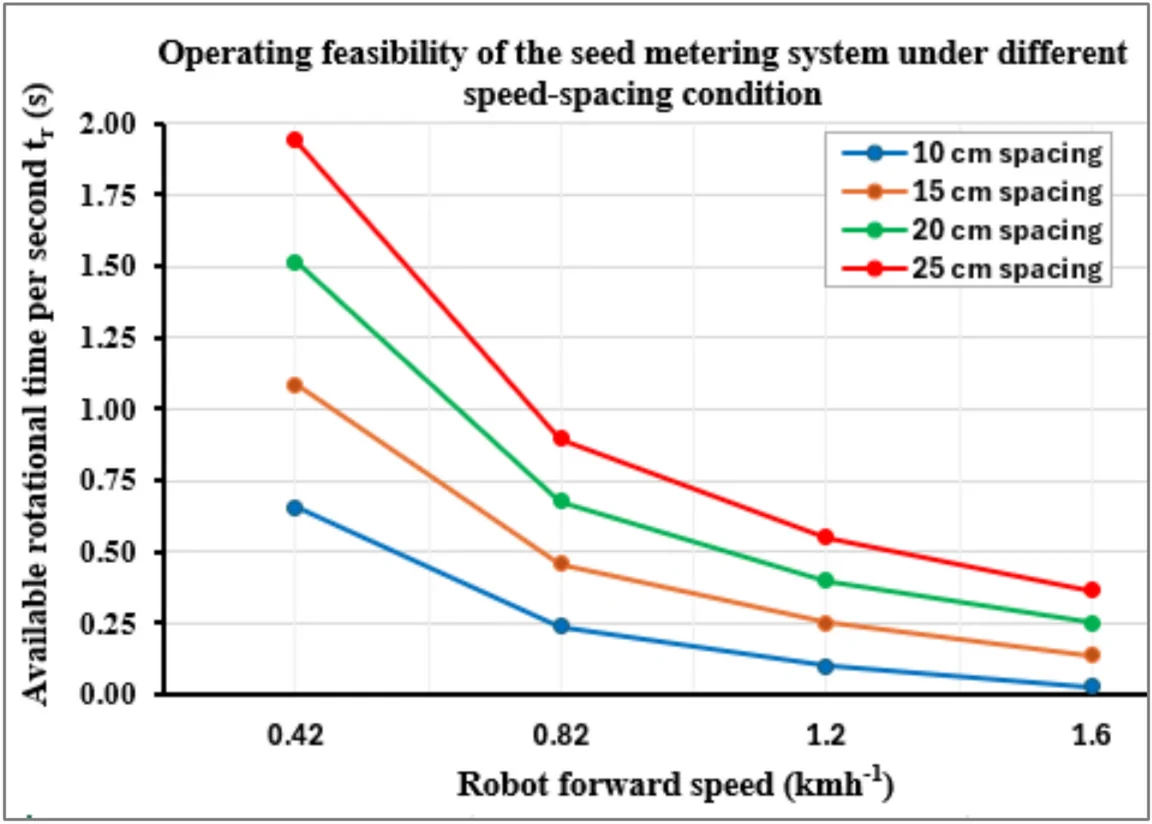
Variation of available rotation time per seed (t_r_) with robot forward speed for different seed spacings.

Seed spacing also played an important role in determining metering feasibility. Larger spacings (20–25 cm) consistently provided longer rotation times because the metering disc required fewer rotations per unit distance. Conversely, smaller spacings (10–15 cm) required higher rotational speeds and therefore shorter release windows, making them more susceptible to potential metering instability at higher robot speeds, as shown in Fig. 14.

Overall, the feasibility analysis indicates that operating the robot within a moderate speed range (approximately 0.42–1.20 km h⁻1) provides the most stable conditions for precision seeding. These findings highlight the importance of coordinating robot travel speed with appropriate seed spacing to maintain adequate dwell time and ensure reliable metering performance in precision planting systems.

### Relationship between robot forward speed and disc rotational speed

The relationship between robot forward speed and seed-metering disc rotational speed $\left({N}_{d}\right)$ was evaluated for four target seed spacings (10, 15, 20, and 25 cm). The results demonstrated that the measured disc speeds closely matched the calculated theoretical values, with deviations generally less than ±1 rpm. This high level of agreement confirms the accuracy of the encoder-based feedback control system used to synchronize the metering motor with robot motion.

**Fig. 15:**
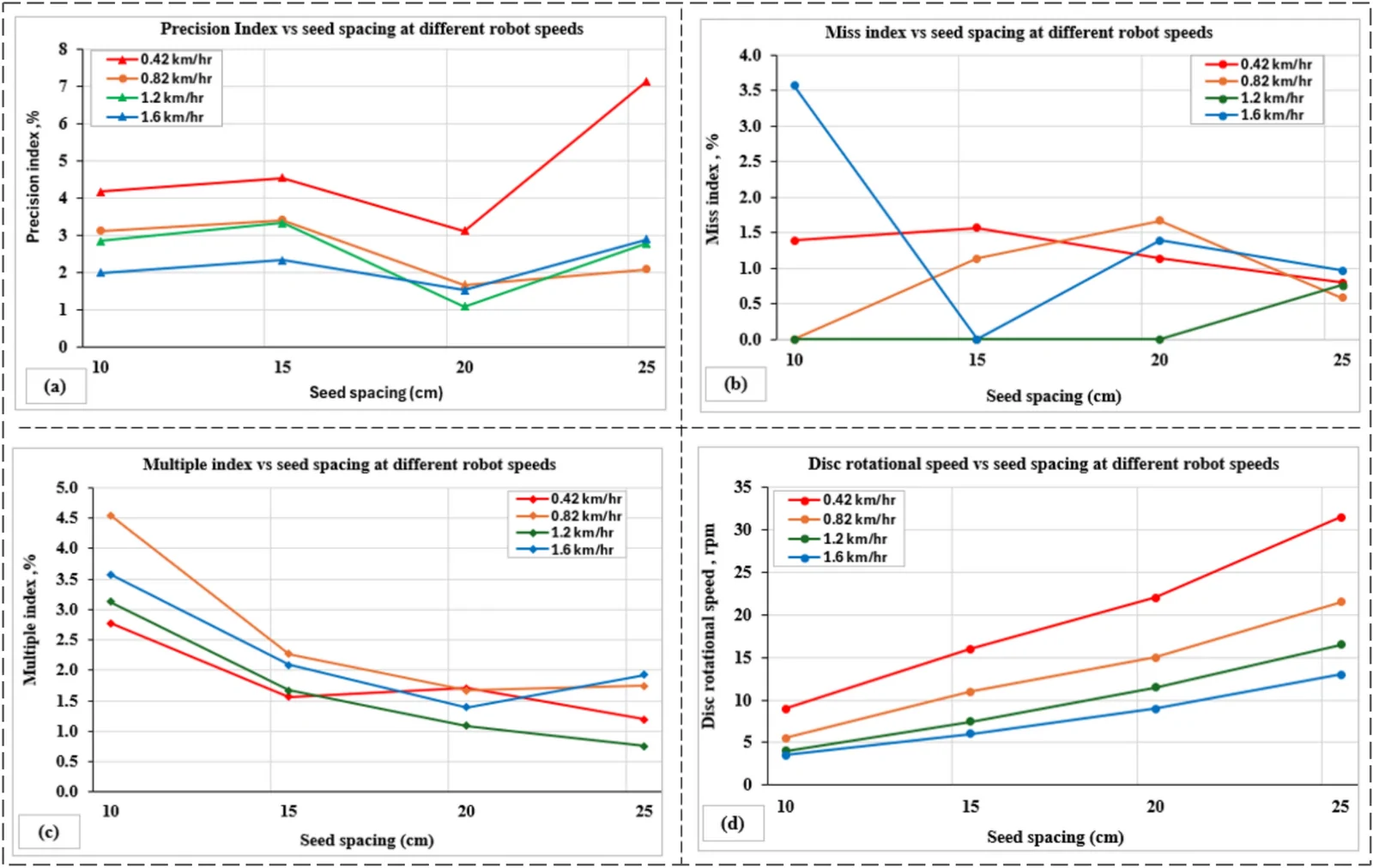
Performance of the electric seed-metering mechanism under different robot speeds and seed spacings (**a**) Precision index, (**b**) miss index, and (**c**) multiple index as a function of seed spacing at four robot forward speeds (0.42, 0.82, 1.2, and 1.6 km h⁻^1^). (**d**) Relationship between robot forward speed and disc rotational speed (N_d_) of the seed-metering mechanism.

Disc rotational speed increased proportionally with robot travel speed for all spacing levels. For instance, at a seed spacing of 10 cm, the disc rotational speed increased from approximately 9 rpm at 0.42 km h⁻1 to about 31.5 rpm at 1.60 km h⁻1. Similar trends were observed for other spacing levels, although larger spacings required lower rotational speeds due to reduced seed release frequency. At the highest speed (1.60 km h⁻1), disc speeds were approximately 21.5 rpm, 16.5 rpm, and 13 rpm for spacings of 15, 20, and 25 cm, respectively, as illustrated in Fig. 15d.

The strong correlation between theoretical and measured values indicates that the encoder feedback system accurately detected the robot forward speed and dynamically adjusted the metering motor to maintain the desired seed spacing. Minor differences between calculated and measured speeds may be attributed to mechanical friction, motor response delays, and small variations in field conditions. Nevertheless, these discrepancies remained minimal, demonstrating stable synchronization between the robot motion and the metering disc rotation.

From an agronomic perspective, the proportional relationship between robot speed and disc rotational speed ensures consistent seed placement across different operating conditions. Accurate disc speed control contributes to improved seed singulation, reduced spacing variability, and lower occurrences of missed or multiple seed drops. Similar trends have been reported in previous studies on electric seed metering systems, where precise synchronization between travel speed and metering frequency was identified as a key factor for achieving high planting accuracy.

These findings confirm that the developed electric seed-metering mechanism provides reliable and precise performance suitable for integration into remotely controlled agricultural robots designed for precision row-crop planting.

### Seed meter performance under different robot speeds and seed spacings

The operational performance of the electric seed-metering device was evaluated under four robot forward speeds (0.42, 0.82, 1.20, and 1.60 km h⁻1) and four target seed spacings (10, 15, 20, and 25 cm). The objective was to determine how the interaction between travel speed and metering disc rotation affects seed placement uniformity in the solar-powered remote-controlled planting robot. The combined influence of robot speed and seed spacing on seed distribution uniformity is illustrated in Fig. 15 through the precision index, which represents the variability of seed-to-seed spacing along the row. Lower precision index values indicate improved uniformity.

As shown in Fig. 15a, seed placement uniformity was highest at moderate robot speeds (0.82–1.20 km h⁻1) combined with intermediate spacing levels (15–20 cm). In contrast, both very low and higher travel speeds resulted in slightly increased variability. This pattern highlights the importance of maintaining synchronization between metering disc rotation and robot ground speed, which directly influences seed release timing in precision planting systems.

#### Effect of robot forward speed on seed delivery performance

Robot forward speed had a noticeable effect on seed placement performance. Key indicators, including seed rate, miss index, multiple index, and precision index were used to evaluate metering quality. Across all tested conditions, the metering device maintained stable seed delivery, although moderate variations in singulation performance occurred with increasing travel speed.

At the lowest robot speed (0.42 km h⁻1), the system demonstrated reliable seed pickup with miss index values ranging from 0.00 to 3.57% and multiple index values between 3.12 and 4.55%, as presented in Fig. 15b. Precision index values ranged from 3.13 to 7.14, indicating acceptable but slightly variable spacing. The increased variability at very low speeds is attributed to reduced disc rotational frequency, which increases the relative impact of minor timing fluctuations during seed release.

When the robot speed increased to 0.82 km h⁻1, overall performance improved noticeably. Miss index values decreased to 0.00–1.56%, and multiple index values remained within 1.56–2.27%. Precision index values were also lower compared with the lowest speed, indicating improved synchronization between disc rotation (6–16 rpm) and forward motion. This operating range appears to provide an optimal balance between mechanical stability and seed pickup dynamics.

At 1.20 km h⁻1, the metering device maintained acceptable singulation performance with miss index values of 0.00–1.67% and multiple index values between 1.09 and 2.08%. However, a slight increase in the precision index suggested increased variability in seed release timing, likely associated with higher disc rotational speeds and dynamic effects.

At the highest speed (1.60 km h⁻1), a modest deterioration in spacing uniformity was observed. Miss index values ranged from 0.76 –1.39%, and multiple index values varied between 0.76 and 1.92%, as seen in Fig. 15c. Although these values remain within acceptable standards for precision planting, the precision index increased (1.52–2.89), indicating greater spacing variability. Such trends are consistent with observations reported by Karayel and Chaudhuri, who found that higher travel speeds increase mechanical vibration and reduce the available synchronization time between seed pickup and release.

Overall, the results indicate that the electric metering system performs most consistently at robot speeds below approximately 1.20 km h⁻1, while higher speeds remain operationally feasible but introduce slightly greater spacing variability.

#### Influence of seed spacing on seed rate and metering stability

**Fig. 16:**
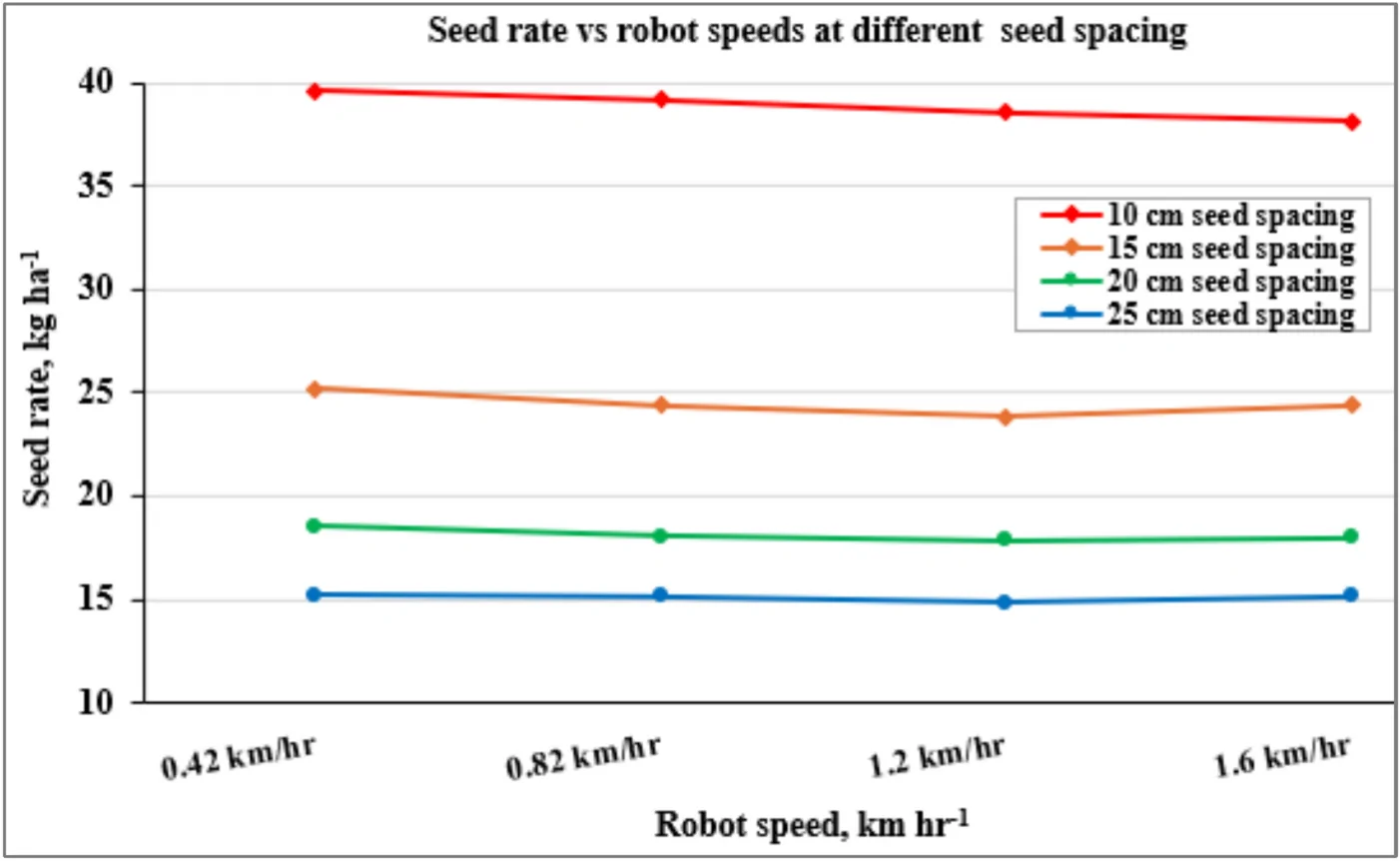
Variation of seed rate (kg ha⁻^1^) with robot forward speed and target seed spacing for the electric seed-metering system.

Seed spacing had a direct influence on seed rate and singulation performance. As illustrated in Fig. 16, increasing the target spacing resulted in a consistent decrease in seed rate across all robot speeds. Average seed rates were approximately 39, 24, 18, and 15 kg ha⁻1 at spacing levels of 10, 15, 20, and 25 cm, respectively. These measured values closely matched theoretical estimates calculated using a thousand-seed weight of 290.06 g, confirming the accuracy of the electric metering disc calibration.

Wider spacing intervals generally improved metering stability. Spacing levels of 20–25 cm produced lower multiple index values and slightly reduced miss index values compared with closer spacings. This behavior can be explained by the reduced seed dispensing frequency required at wider spacing intervals, allowing more stable seed pickup and release from the metering holes.

The relatively small variation in seed rate across robot speeds indicates that the electronic motor control system effectively compensated for velocity changes. The close overlap of seed-rate curves across speeds suggests that synchronization between disc rotation and robot forward motion remained stable. Similar behavior has been reported in electronically controlled seed metering systems^11^, which emphasized the importance of electronic control in maintaining accurate seed delivery under variable field conditions.

#### Seed spacing accuracy at different operating conditions

Seed spacing accuracy represents one of the most critical performance indicators for precision planting systems. The evaluation was conducted using a randomized complete block design considering robot speed and target spacing as experimental factors. The results showed that the electric seed-metering system maintained high spacing accuracy across all operating conditions.

Spacing accuracy ranged from 98.08% to 98.80%, demonstrating excellent consistency in seed placement. At the lowest robot speed (0.42 km h⁻1), spacing accuracy varied between 98.28% and 98.80%, with minimal spacing error below 0.30 cm. Increasing the robot speed to 0.82 km h⁻1 produced similar accuracy values (98.60–98.70%), confirming effective synchronization between disc rotation and ground motion.

**Fig. 17:**
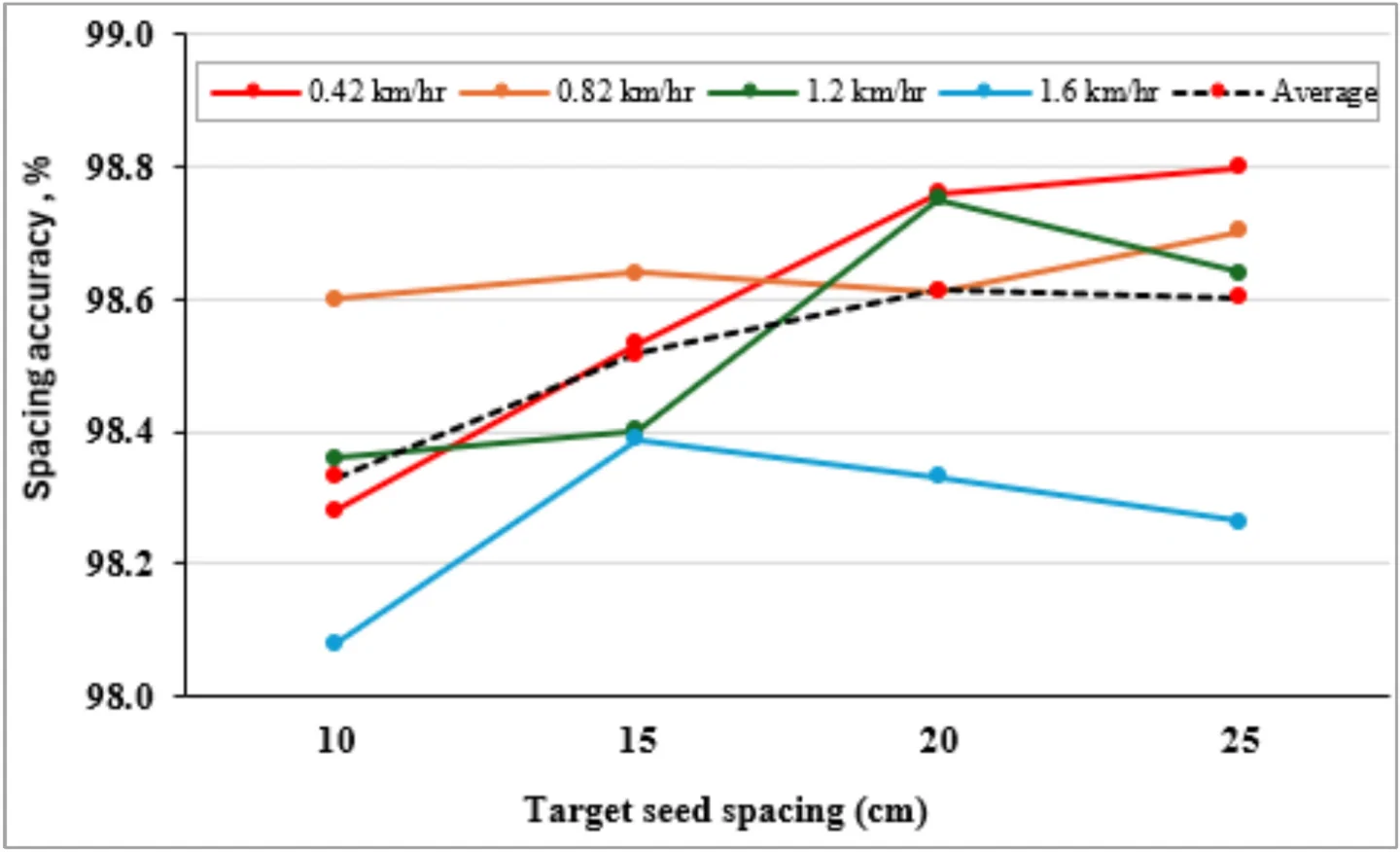
Spacing accuracy (%) of the electric seed metering device at different target seed spacings (10- 25 cm) and robot forward speeds.

At the intermediate speed of 1.20 km h⁻1, spacing accuracy remained high (98.40–98.75%), although slightly higher coefficients of variation were observed at certain spacing levels. The highest speed (1.60 km h⁻1) resulted in the lowest accuracy values (98.08–98.39%), reflecting increased mechanical vibration and reduced seed stabilization time (Fig. 17).

The experimental results of an electric seed-metering system demonstrate that a slight improvement in spacing accuracy was observed as the target spacing increased from 10 to 25 cm. Wider spacing intervals provide longer time intervals between seed releases, reducing the effect of transient timing errors. Similar trends have been reported in recent studies on electric-drive seed metering systems^17,32,33^, which observed that larger spacing intervals improve seed placement precision by reducing dispensing frequency. Conversely, smaller spacings (10–15 cm) required higher dispensing frequency, leading to slightly higher variability. These observations agree with previous findings reported^11,33^.

#### Statistical analysis of metering performance

A two-factor analysis of variance ANOVA with replication (4 × 4 design, n = 5) was performed to quantify the effects of robot speed and seed spacing on spacing accuracy and metering performance. The results revealed that both factors significantly affected spacing accuracy (p < 0.0001), while their interaction was also highly significant (Table 2).

**Table 2:** Two-way ANOVA results showing the effects of robot forward speed, target seed spacing, and their interaction on seed spacing accuracy.

Source of variation	SS	df	MS	F-value	P-value	Significance
Robot speed	0.534	3	0.178	174.51	<0.0001	***
Seed spacing	0.345	3	0.115	112.75	<0.0001	***
Speed x spacing	0.167	9	0.0185	18.20	<0.0001	***
Error	0.0654	64	0.00102	–	–	–
Total	1.1114	79	–	–	–	–

***indicates statistical significance at p < 0.001.

Robot speed exhibited the strongest influence on spacing accuracy (F = 174.51), indicating that accuracy gradually decreased as forward speed increased. Seed spacing also had a significant effect (F = 112.75), with wider spacing levels producing slightly improved accuracy due to reduced seed release frequency.

Further statistical analysis of performance indicators showed that the miss index was not significantly affected by either robot speed or seed spacing. This indicates that the seed pickup mechanism reliably deposited at least one seed under all tested conditions. Similar findings were reported by Jasa and Dickey^34^ in earlier planter performance studies.

In contrast, the multiple index was strongly influenced by robot speed (p < 0.001) and moderately affected by seed spacing (p < 0.05). Higher speeds increased the probability of multiple seed discharge due to reduced seed filling time and increased dynamic disturbance inside the metering unit.

The precision index was also significantly affected by robot speed (p < 0.05), confirming that higher forward speeds increase spacing variability. These findings are consistent with previous research^35–37^ indicating that planting speed is a critical factor influencing seed placement accuracy in both mechanical and electronically controlled metering systems.

#### Overall performance of the electric seed meter

The electric seed-metering device demonstrated stable and reliable performance across the tested operating conditions. Miss index values remained below 2% in most treatments, indicating consistent seed pickup and effective seed-disc interaction. Multiple index values were generally below 2.5%, confirming effective singulation and minimal seed doubling.

The precision index ranged between approximately 1.0 and 7.1%, depending on travel speed, with the most consistent spacing observed at moderate speeds. The close agreement between theoretical and measured seed rates further confirmed that the electronic motor control system successfully synchronized disc rotation with robot forward motion.

Based on the combined analysis, the optimal operating range for the metering system was achieved at robot speeds between 0.82 and 1.20 km h⁻1 and seed spacings between 15 and 20 cm. Within this region, the system achieved minimal spacing variability, low miss rates, and stable seed discharge.

These findings confirm that the electric seed-metering mechanism provides the accuracy and stability required for precision planting. The results demonstrate the suitability of the proposed solar-powered robotic planter for precision agriculture applications requiring consistent seed placement and reliable spacing control.

Overall, the results demonstrate that robot forward speed is the dominant operational factor influencing seed metering performance, while target seed spacing plays a secondary role in determining spacing uniformity. Maintaining moderate operating speeds between 0.82 and 1.20 km h⁻1 provided the most stable metering performance, minimizing spacing variability while maintaining high singulation accuracy. In contrast, higher speeds remain operationally feasible but introduce slightly greater spacing variability.

### Calibration of the seed depth adjustment unit.

The seed depth adjustment unit was calibrated to determine the relationship between DC motor rotations and seed opener penetration depth. The mechanism consisted of a 3600 rpm DC motor, a 1:60 reduction gearbox, and a lead screw (6 mm diameter, 1 mm pitch) that converted rotational motion into linear displacement. The theoretical seed depth was estimated using ${D}_{cm}={R}_{m}/600$, where ${R}_{m}$ represents the motor rotations.

Experimental measurements closely matched the theoretical values (Table 3), with deviations within ±0.06 cm, indicating high mechanical precision.

**Table 3:** Theoretical and experimental seed depths at different motor rotation levels.

Motor rotations (rev)	Theoretically calculated depth (cm)	Experimentally measured depth (cm)	Deviation (mm)
0	0.000	0.00	0.00
500	0.833	0.883	0.05
1000	1.667	1.627	-0.04
1500	2.500	2.56	0.06
2000	3.333	3.283	-0.05
2500	4.167	4.207	0.04
3000	5.000	4.94	-0.06
3500	5.833	5.883	0.05
4000	6.667	6.627	-0.04
4500	7.500	7.56	0.06

**Fig. 18:**
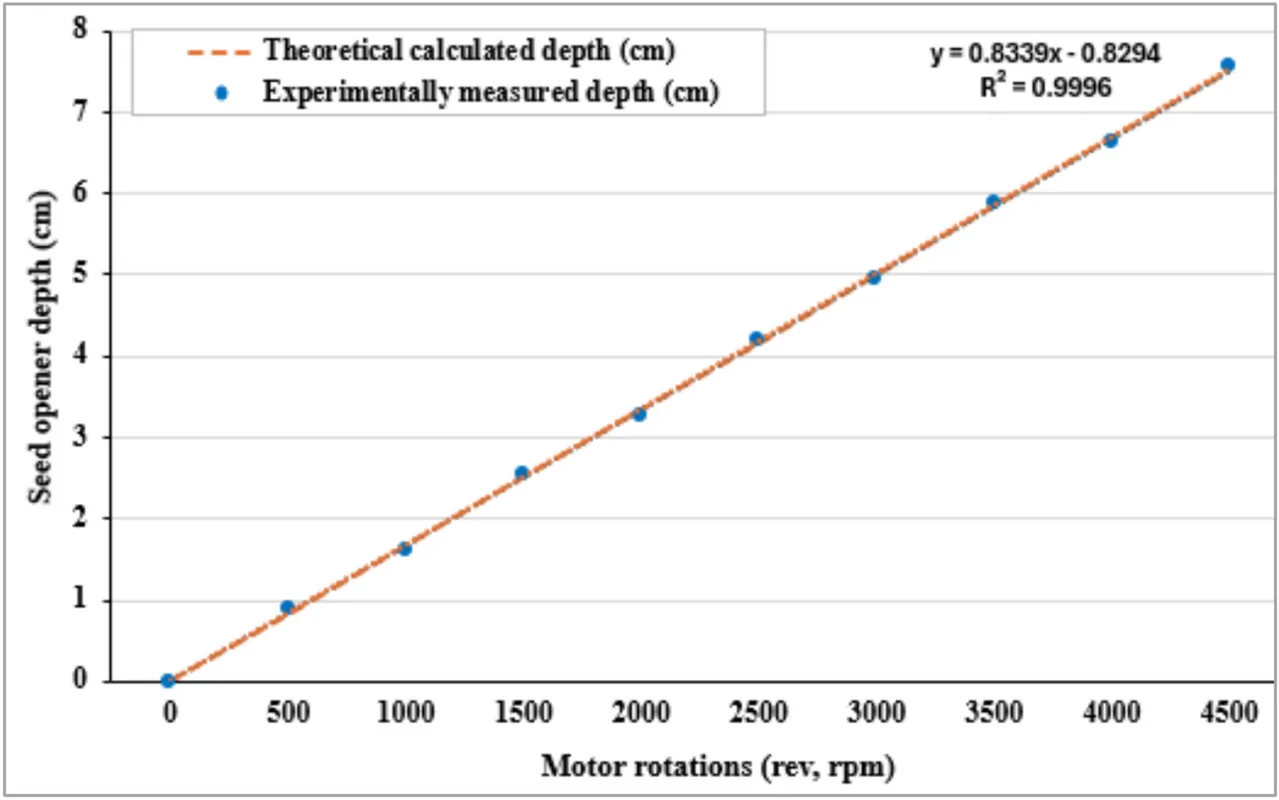
The calibration curve shows the relationship between motor rotations and seed opener penetration depth.

As illustrated in Fig. 18, a strong linear relationship was observed between motor rotations and seed depth, with a coefficient of determination (R2 = 0.9996). Minor deviations were attributed to soil resistance and friction within the lead screw mechanism.

Overall, the calibration results demonstrate that the DC motor–gearbox–lead screw assembly provides accurate and fine-resolution control of seed placement depth. The calibrated depth–rotation relationship was integrated into the robot control algorithm, enabling automatic depth adjustment during precision planting operations. Compared with conventional manually adjusted seed depth mechanisms, the proposed electronically controlled system offers greater precision, repeatability, and adaptability, thereby improving the overall planting performance of the solar-powered remote-controlled seeding robot.

### Power consumption and solar power supply ratio

**Fig. 19:**
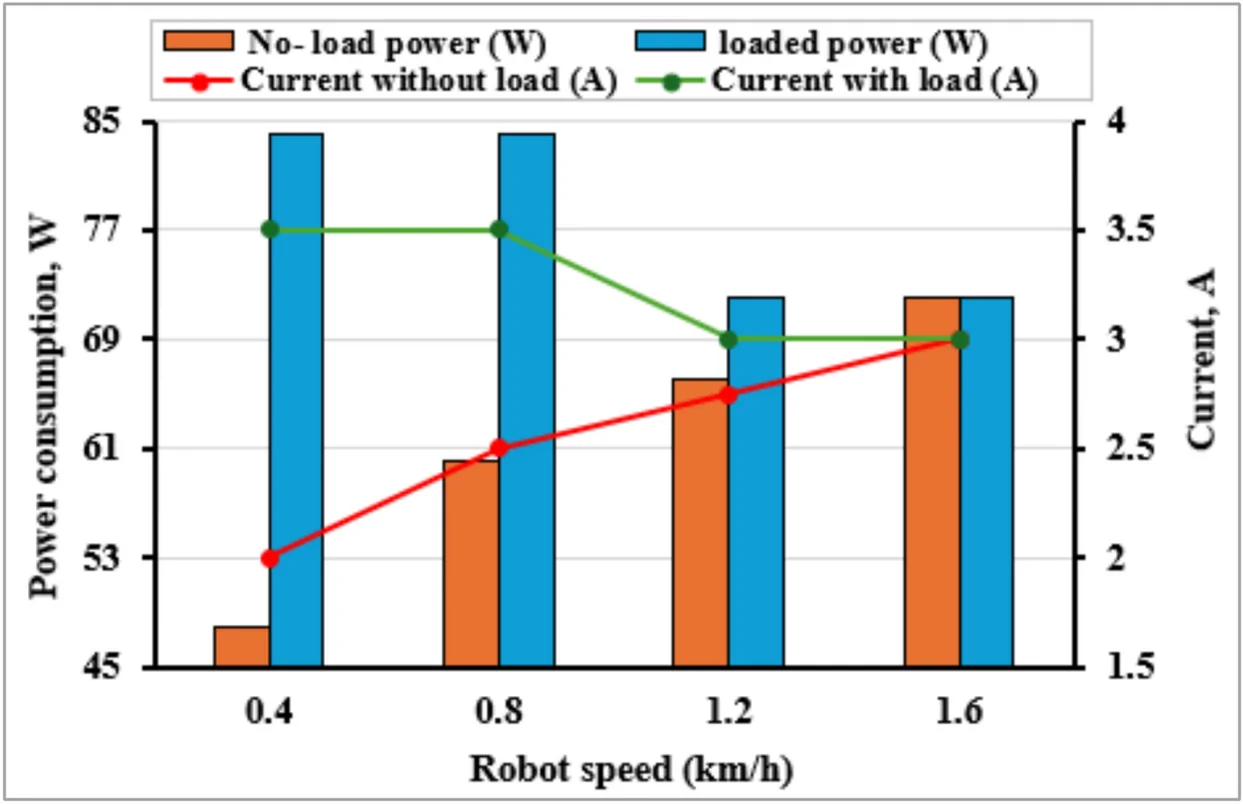
Power consumption of the agricultural robot as a function of forward speed under no-load and loaded conditions.

The power consumption of the solar-powered remote-controlled agricultural robot was evaluated at four forward speeds under both no-load and loaded conditions. Under no-load operation, the current increased from 2.0 A at 0.42 km h⁻1 to 3.0 A at 1.6 km h⁻1, resulting in an increase in power consumption from 48 W to 72 W (Fig. 19). Consequently, the estimated battery operating time decreased from 4.0 h to 2.67 h as the speed increased.

The solar power supply ratio, defined as the ratio of the available photovoltaic power to the measured robot power consumption, ranged from 111.1% to 166.7% under no-load conditions. These values indicate that the photovoltaic system was capable of supplying the robot’s entire electrical demand while providing surplus energy for battery charging at lower operating speeds.

Under loaded conditions, the current demand ranged between 3.0 to 3.5 A, corresponding to power consumption between 72 and 84 W. The estimated operating time varied from 2.29 to 2.67 h, while the SPSR ranged from 95.2% to 111.1%, At moderate speeds, the photovoltaic system supplied nearly all the required electrical power, whereas at the highest power demand, the battery complemented the photovoltaic supply when necessary.

Overall, robot speed significantly influenced energy consumption, and the solar power supply ratio (p < 0.05), with moderate speeds (1.2–1.6 km h⁻1) provided a favorable balance between operational performance, battery endurance, and photovoltaic power contribution. These results demonstrate that the developed solar-powered robotic planting system can effectively satisfy its electrical power requirements while reducing dependence on battery energy during field operation.

### Carbon emission reduction analysis

The developed solar-powered robot consumed 0.048–0.084 kWh under the tested operating conditions and produced no direct on-site CO₂ emissions during operation. In contrast, small diesel-powered planting machines typically consume 0.6–0.8 L h⁻1, corresponding to approximately 6–8 kWh and 1.6–2.1 kg CO₂ h⁻1^38–40^. These results demonstrate the substantially lower energy demand and elimination of direct exhaust emissions achieved by the proposed solar-powered system. However, because the prototype robot and conventional planting machines differ in field capacity, a direct environmental comparison should be normalized on an area basis (kg CO₂ ha⁻1). Such an analysis will be conducted in future studies after large-scale field-capacity evaluations.

## Conclusions

This study developed and evaluated a solar-powered remote-controlled robot for precision seed planting, integrating a photovoltaic energy system, electric seed-metering mechanism, and electronic control unit to achieve accurate seed placement with reduced dependence on fossil-fuel-powered machinery. Experimental results demonstrated high seed placement accuracy across all operating conditions, with spacing accuracy ranging from 98.08% to 98.80%, while the miss and multiple indices remained below 2% and 2.5%, respectively, indicating reliable seed singulation and uniform seed distribution.

Robot forward speed significantly influenced metering performance, with optimal operation observed at 0.82–1.20 km h⁻^1^, where seed placement uniformity was highest. Wider target spacings (20–25 cm) further improved distribution consistency by reducing seed dispensing frequency and stabilizing seed pickup. The calibrated seed-depth adjustment unit showed a highly linear relationship between motor rotations and opener depth (R^2^ = 0.9996), confirming precise and repeatable depth control.

Energy analysis revealed low power consumption (48–84 W) and a solar power supply ratio ranging from 95% to 167%, indicating that the photovoltaic system could adequately support the robot’s energy demand under the tested operating conditions. Compared with conventional diesel planting equipment, the system produces zero direct on-site CO₂ emissions during operation. These findings demonstrate the feasibility of solar-powered agricultural robotics as an energy-efficient and environmentally sustainable solution for precision planting.

## Recommendations for future work

Future work should focus on integrating advanced navigation systems (e.g., RTK-GNSS or machine vision) to enable the developed robotic platform to operate fully autonomously under field conditions. Future work will include a comprehensive environmental assessment based on planted-area-specific emissions (kg CO₂ ha⁻1), incorporating field-capacity measurements to enable a rigorous comparison with conventional planting systems under large-scale operating conditions. Expanding the robot to multi-row planting could increase field capacity for large-scale applications. Further measurements of solar irradiance, photovoltaic output power, battery charging efficiency, and energy flow under field conditions to validate the energy performance of the solar-powered robotic system experimentally.

## Data Availability

All available data generated or analyzed during this study have been included in this published article. No additional datasets were generated or analyzed during the current study.
